# Elemental profiling and genome-wide association studies reveal genomic variants modulating ionomic composition in *Populus trichocarpa* leaves

**DOI:** 10.3389/fpls.2024.1450646

**Published:** 2024-11-28

**Authors:** Raphael Ployet, Kai Feng, Jin Zhang, Ivan Baxter, David C. Glasgow, Hunter B. Andrews, Miguel Rodriguez, Jin-Gui Chen, Gerald A. Tuskan, Timothy J. Tschaplinski, David J. Weston, Madhavi Z. Martin, Wellington Muchero

**Affiliations:** ^1^ Biosciences Division, Oak Ridge National Laboratory, Oak Ridge, TN, United States; ^2^ Center for Bioenergy Innovation, Oak Ridge National Laboratory, Oak Ridge, TN, United States; ^3^ Donald Danforth Plant Science Center, St. Louis, MO, United States; ^4^ Chemical Sciences Division, Oak Ridge National Laboratory, Oak Ridge, TN, United States; ^5^ Radioisotopes Science and Technology Division, Oak Ridge National Laboratory, Oak Ridge, TN, United States

**Keywords:** ionomics, GWAS, plasma-mass spectrometry, neutron activation analysis, laser-induced breakdown spectroscopy, molybdenum transporter, ion homeostasis, *Populus*

## Abstract

The ionome represents elemental composition in plant tissues and can be an indicator of nutrient status as well as overall plant performance. Thus, identifying genetic determinants governing elemental uptake and storage is an important goal for breeding and engineering biomass feedstocks with improved performance. In this study, we coupled high-throughput ionome characterization of leaf tissues with high-resolution genome-wide association studies (GWAS) to uncover genetic loci that modulate ionomic composition in leaves of poplar (*Populus trichocarpa*). Significant agreement was observed across the three ionomic profiling platforms tested: inductively coupled plasma-mass spectrometry (ICP-MS), neutron activation analysis (NAA) and laser-induced breakdown spectroscopy (LIBS). Relative quantification of 20 elements using ICP-MS across a population of 584 genotypes, revealed larger variation in micro-nutrients and trace elements content than for macro-nutrients across genotypes. The GWAS performed using a set of high-density (>8.2 million) single nucleotide polymorphisms, identified over 600 loci significantly associated with variations in these mineral elements, pointing to numerous uncharacterized candidate genes. A significant enrichment for genes related to ion homeostasis and transport was observed, including several members of the cation-proton antiporters (CPA) family and MATE efflux transporters, previously reported to be critical for plant growth and fitness in other species. Our results also included a polymorphic copy of the high-affinity molybdenum transporter MOT1 found directly associated to molybdenum content. For the first time in a perennial plant, our results provide evidence of genetic control of mineral content in a model tree species.

## 1 Introduction

A multi-omics profile includes representation by four biochemical pillars, i.e., the transcriptome, proteome, metabolome, and ionome, which collectively describe, in part, the molecular functioning of a living organism. The study of the transcriptome (Klepikova et al., 2016; Zhu and Wang, 2000), proteome (Cánovas et al., 2004; Abraham et al., 2015; Mergner and Kuster, 2022; Castillejo et al., 2023), and metabolome (Fiehn et al., 2000; Tschaplinski et al., 2019) have been undertaken in substantial detail in many organisms, but the ionome investigations are just emerging (Rea, 2003; Baxter, 2009; Singh et al., 2013) and many of the genes and gene networks involved in ionome regulation remain largely unknown. The ionome includes all metal, metalloid, and nonmetal elements present in an organism (Salt et al., 2008), including all macro- and micro-nutrients. In plants, macro-nutrients (N, P, K, Ca, S, Mg) are required in large amounts, whereas micro-nutrients and trace elements (Ni, Mo, Cu, Zn, Mn, B, Fe, Cl) are usually required in smaller amounts (Williams and Salt, 2009). Macro-nutrients play many pivotal roles in plant development. For example, K and Ca are the most abundant free cations in plant cells (Sardans and Peñuelas, 2021), where K regulates activity of numerous enzymes, playing a role in photosynthesis and biosynthetic pathways and is involved in osmo-regulation (Cui and Tcherkez, 2021). It allows plant cells to regulate their water potential which in turns allows plants to control their stomatal aperture, and uptake water and nutrients, contributing to development and adaptation to water deficit conditions (Cakmak, 2005; Mostofa et al., 2022). For most plant species, abundance of K was shown to greatly improve primary and secondary growth (Sardans and Peñuelas, 2021). Ca is also involved in a range of developmental processes, including plant cell walls formation through its role in pectins organization and as an intra-cellular messenger that allows plants to respond to environmental stimuli. Another essential macronutrient, P, is incorporated in numerous biomolecules, such as nucleic acids and phospholipids playing structural roles and in signaling via protein phosphorylation. In addition to these major elements, micronutrients play key roles as enzymatic co-factors and signaling molecules. While macro-nutrients are generally growth limiting factors in case of their deficiency, a severe impact of depletion of other micronutrients, such as Zn and Mo, has also been documented, leading to impaired growth and poor resistance to stresses (Arnon and Stout, 1939; Fageria et al., 2002). Mo is an example of micronutrient required in trace amounts, serving as a co-factor for numerous molybdoenzymes that catalyze oxidation–reduction reactions essential for nitrogen and sulfur metabolism (Tejada-Jiménez et al., 2013).

Other elements such as Na, Co, Al, and Se are also known to have biological functions in plants (Williams and Salt, 2009). For example, Na, a non-essential element for most plants, is also one of the most abundant ions in plant tissues (Subbarao et al., 2010). While it is mostly known for its toxic effect and negative impact on growth at high concentrations (Adams and Shin, 2014; Maathuis, 2014), it was shown that amendment with Na could at least partially alleviate K depletion through its unspecific function as a monovalent cation, having a positive effect on growth and resistance to water deficit in different species (Epron et al., 2012; Erel et al., 2014; Favreau et al., 2019; Tomemori et al., 2002; Wakeel et al., 2011, 2010). This highlights that different ions can have overlapping functions, sometimes competing for transport or simply showing strong interactions at chemical, physiological and/or genetic levels (Baxter, 2015; Bouain et al., 2019).

In addition to the uptake, compartmentalization of these elements is a key regulatory process. Ion transporters are of central importance, making these proteins the primary focus of most research involved in characterizing the underlying genetic components of ionome regulation in plants. However, ion homeostasis is often regulated by a complex cooperation of multiple genes and the existence of different levels of interactions between mineral elements make ionome investigation challenging for conventional forward genetics approaches (Mäser et al., 2001).

Different techniques are commonly used to determine the elemental composition. Rapid multi-elemental microanalysis of different types of sample matrices (solid, liquid, gases, and aerosols) was previously demonstrated using the laser induced breakdown spectroscopy (LIBS) technique (Cremers and Radziemski, 2017; Radziemski and Cremers, 2020). The detection of most elements from these matrices was in the parts-per-million (ppm) range, with the resulting spectrum consisting of emission peaks that are representative of all the elements present in the sample. Another method, neutron activation analysis (NAA) is a nuclear analytical trace element metrology technique that utilizes neutron irradiation to induce radioactivity that is then emitted and detected to reveal the number and kind of trace elements composing a sample. Inductively coupled plasma mass spectrometry (ICP-MS) is a versatile trace element quantification method that is well-suited to large sample sets because it can detect many of the elements simultaneously. In contrast to LIBS and NAA, sample dissolution is required for ICP-MS, which is often carried out in acid using microwave-assisted digestion.

Watanabe et al. (2007) used NAA to analyze 44 elements in 2,228 leaf samples from 670 species of terrestrial plants spread across 138 distinct families. They showed that over 25% of the total variation in leaf elemental composition could be assigned to the phylogenetic family level (Watanabe et al., 2007), suggesting inheritance and a genetic component to variation in the ionome. Similar observations were made by Neugebauer and colleagues when surveying many studies performed in angiosperms (Neugebauer et al., 2018). The fact that these phylogenetic effects explained not only variations in the macronutrients N and P, but also other micronutrients and trace elements, support the hypothesis that a large proportion of the ionome in plants is under tight genetic control. While several studies have successfully captured variations of the ionome across populations of dicots (*Arabidopsis*, potato, and Cassava; Lahner et al., 2003; Baxter et al., 2012; Yin et al., 2022) and monocots (rice and maize; Yang M. et al., 2018; Wu et al., 2021), very little is known about variations in the mineral content of populations of perennial woody species (Singh et al., 2013).

The use of *Populus* spp. (poplars, aspens, and cottonwoods) is increasing globally as demand for lignocellulosic biomass increases (Clifton-Brown et al., 2019). The *Populus* genus, consisting of woody perennial species that occupy diverse habitats ranging from the tropics to the sub-arctic, are dominant members of riparian systems and are important pioneer species within boreal forests. *Populus* is largely undomesticated with natural populations exhibiting a high degree of genetic and phenotypic trait variability. Variation in mineral accumulation in natural populations of poplars and the genetic determinants of variation remain mostly unknown. As an alternative to conventional genetic screens, genome-wide associations studies (GWAS) have been instrumental for discovering genetic determinants of complex traits in many plant species, including poplar (Tuskan et al., 2018a; Gupta et al., 2019). By precisely linking genomic mutations to population-wide phenotypic variation, GWAS performed in poplar pointed to regulatory loci for a variety of phenotypes, including phenology (Evans et al., 2014), lignin biosynthesis (Xie et al., 2018), metabolite biosynthesis (Zhang et al., 2018) and disease resistance (Muchero et al., 2018), leading to the discovery of new targets for genetic engineering. GWAS approaches have also been successfully applied to the discovery of regulators of the ionome in annual plants (Lahner et al., 2003; Baxter et al., 2010; Forsberg et al., 2015; Yang M. et al., 2018; Wu et al., 2021; Yin et al., 2022).

In most cases, the main challenge for performing GWAS is to have a cost-effective, sensitive, and scalable method to analyze the phenotype of interest. For ionomics, the three most common methods for high-throughput elemental analysis are: 1) atomic absorption spectroscopy (AAS), 2) inductively coupled plasma-optical emission spectroscopy (ICP-OES), and 3) ICP-mass spectrometry (ICP-MS). In addition to these, LIBS has also been used as a novel high-throughput technique to determine the ionome of poplar leaves (Kaiser et al., 2012; Krajcarova et al., 2013; Martin et al., 2017), while X-ray fluorescence spectroscopy (XRF) has been used for elemental analysis of multiple plant samples (Salt et al., 2008).

In this work, we systematically assessed the sensitivity, and scalability of multiple platforms for characterizing the elemental profile of a large number of poplar leaf samples. Using ICP-MS, we then profiled the ionome of a large population of natural variants of black cottonwood poplar (*Populus trichocarpa*) grown in a common garden. This allowed investigating to what degree genetic variation impacts poplar leaf elemental composition. Then, the profiles of 19 individual mineral elements were used as quantitative traits in a GWAS, to identify the genetic determinants underlying leaf elemental composition in poplar. This approach identified several known key regulators of elemental accumulation in plant tissues and several novel candidate genes that play potential critical roles in ion homeostasis and in the interaction between mineral elements, providing new targets for engineering traits such as improved growth and resistance to biotic and abiotic stressors.

## 2 Material and methods

### 2.1 Plant material and sample collection

A population of 1,089 black cottonwood genotypes (*P. trichocarpa*) was assembled from native stands to encompass the central portion of the natural range of the species, stretching from 38.8° to 54.3° N latitude from California, USA, to British Columbia, Canada (**Supplementary Figure S1A**). Establishment of the common garden, growth conditions, and site maintenance have been described by Muchero et al (Muchero et al., 2015). In this study, leaf samples for ionomic profiling were collected from 4-year-old trees, during the growing season, in July 2012, from a field located in Clatskanie, Oregon, USA (46°6′11″N 123°12′13″W). The field site was located in a protected alluvial floodplain containing a uniform Wauna-Locoda silt loam soil area characterized by an acidic pH, in Columbia County, Oregon. The elemental profile of soil samples, collected at a depth of 30 centimeters at four different randomly picked locations within the field site, was obtained using LIBS (**Supplementary Figure S2**; **Supplementary Table S1**). A subset of 584 out of the 1,089 *P. trichocarpa* genotypes were represented in this sampling. These genotypes were randomly selected to represent the geographical distribution of the population (**Supplementary Figure S1B**). A single fully mature (LPI 7-9) leaf on the south side of the tree exposed to full sunlight conditions was removed from the tree within a 6-hour window centering on solar noon and immediately frozen under dry ice before processing.

### 2.2 Sample preparation

Initially, 25 poplar samples and 5 NIST standard reference materials were chosen for LIBS and NAA. The 25 poplar leaf samples randomly chosen among the 584 genotypes used in this study were finely ground to 40 μm particle size using a mortar and pestle. To obtain consistent pellets that would be robust enough to withstand the laser energy that is used to excite the samples in LIBS, 75 mg of the ground powder for each sample was weighed then humidified by equilibrating the weighed samples in a chamber with open beakers of water for 12 hours. The humidified powder was pelletized for 3 min at pressures of 2000 psi in a Carver press with a 6 mm stainless steel (grade 440 C) evacuable die (Martin et al., 2010). Then, pellets were used for NAA and LIBS. Soil samples were pelletized following the same procedure using a 13 mm evacuable die at a pressure of 15000 psi without prior grinding. Five NIST standard reference materials, including spinach leaves, orchard leaves, tomato leaves, pine needle, and bovine liver, were used as comparators. Two sets of 30 calibration samples were prepared. One set was used to perform the LIBS measurements and the second set was used to do the NAA experiments. No chemical treatment was required for either technique. The detection of the 9 elements (Ca43, Mg25, Mn55, Na23, K39, Cu, Fe58, Cl35, and Al27) using LIBS technique were correlated to the NAA results and used to construct a regression model for these elements.

### 2.3 Laser induced breakdown spectroscopy

Previously, LIBS spectra were correlated to NAA concentrations for Ca, Mg, Mn, Al, Cu, and K (Martin et al., 2015, 2017). In this study, additional elements have been identified using LIBS and correlated to the standard NAA technique (using the function *lm* implemented in R). These elements were Na23, Fe57, Cr52, Co, As75, Zn66, Br80, Rb85, Sb119, La139, Ce140, and Sm150. The excitation laser was a Q-switched Nd : YAG laser with frequency doubled output wavelength of 532 nm and a laser energy of 45 mJ (Martin et al., 2010). A displacement laser (650 nm) was employed to maneuver the vertical position of the upper surface of the sample, ensuring that the excitation laser beam remained focused and that the plasma was optimally located with respect to the collection lenses. The light emitted by the plasma at the focal volume was collected by a set of fused silica lenses and re-focused into a low O-H silica fiber of internal diameter 100 μm. This optical fiber delivered the light from the plasma spark to an Echelle spectrometer (Catalina Scientific model SE 200). A high-order dispersion module was used to separate the white light formed in the plasma into respective wavelengths in the large wavelength range (190-800 nm) with a wavelength resolution of 0.06 nm in the optimized range of 300-500 nm. An intensified charge coupled detector (ICCD) (Andor Technologies, Belfast, Northern Ireland) was used to convert the optical signal into a digital signal. The data for all pellets were collected using a 1 μs delay and 10 μs emission detection window. The laser was operated at 10 Hz repetition rate, which allowed data collection every 100 ms. For each pellet, 50 laser pulses were executed and the 50 spectra were averaged to get the elemental peak heights and calculate the concentrations of each element.

### 2.4 Neutron activation analysis

The NAA of leaf samples was described in a previous study (Martin et al., 2017), and a similar protocol was used for this study, with minor modifications. The irradiations were made in the pneumatic tube PT-1, for a duration of 300 s, and counting followed 5-7 days later. Measurement of the samples on high-purity germanium (HPGe) detectors occurred both at 30 mm shelf height and sometimes directly on the endcap. The detectors were ORTEC GEM30P4 p-type HPGe with aluminum end window and digital signal processing was accomplished using the Mirion Lynx operated in loss-free counting mode employing the peak add back method. Because comparator NAA was employed throughout this work, the effects of efficiency calibration, coincidence summing, gamma-ray attenuation, and the uncertainty of nuclear parameters, such as cross sections and gamma-ray branching ratios, were assumed to be constant. Since NAA analyze the whole pellet, a single measurement was done per genotype.

### 2.5 Inductively coupled plasma mass spectrometry

Leaf samples from the 584 P*. trichocarpa* genotypes were ground using a mortar and pestle, oven-dried and analyzed for ionomic composition using ICP-MS. In total, 20 elements were profiled, including aluminum (Al27), arsenic (As75), boron (B11), cadmium (Cd111), calcium (Ca43), cobalt (Co), copper (Cu), iron (Fe57), magnesium (Mg25), manganese (Mn55), molybdenum (Mo), nickel (Ni60), phosphorus (P31), potassium (K39), rubidium (Rb85), selenium (Se82), sodium (Na23), strontium (Sr88), sulfur (S34), and zinc (Zn66), following a protocol established by Ziegler et al. (2013). For each sample, 75mg of powder was digested overnight in 2.5 mL HNO_3_ containing 20 parts per billion (ppb) indium as an internal standard, following the protocol described in Ziegler et al. (2013). Following a dilution, concentration of the 20 elements was measured using an Elan 6000 DRC-e mass spectrometer (Perkin-Elmer SCIEX) connected to a PFA microflow nebulizer (Elemental Scientific) and Apex HF desolvator (Elemental Scientific). One measurement per sample per genotype was done. For subsequent analyses, the quantifications were converted to total element concentration, and isotope numbers are not represented.

### 2.6 GWAS and candidate gene identification

The GWAS were performed with elemental composition as the phenotypic variable by using the analytical platform described by Zhang et al (Zhang et al., 2018). The single nucleotide polymorphism (SNP) and indels were called from whole genome sequencing (short reads) for 917 wild accessions of *P. trichocarpa* as described by Evans et al (Evans et al., 2014). and using the *P. trichocarpa* v3.0 reference genome. The dataset used in this study is available at http://bioenergycenter.org/besc/gwas/. To assess genetic control, we used the Efficient Mixed-Model Association (EMMA) algorithm implemented in the EMMAX software (Kang et al., 2010) with kinship as the correction factor for genetic background effects to compute genotype to phenotype associations (univariate) based on 8,253,066 SNP variants with minor allele frequencies ≥0.05 identified from whole-genome resequencing. The phenotypic data consisted in one measurement per genotype for a subset of the population (584 genotypes). For all phenotypes, the top 100 most significant SNPs (lowest *p*-value) with -log10(*p*) >6 were retained for further analysis. For each significant SNP, the flanking genes detected in a 20-kb window centered around the SNP position, were considered as potential candidate genes for that locus. Results were visualized as networks using Cytoscape v3.9.1.

Co-expression data was obtained by calculating pairwise Pearson correlations between gene profiles across 8 tissues of *P. trichocarpa* (total of 23 RNAseq samples) from previously published data (SRA accession PRJNA320431; Shi et al., 2017). RNAseq data were retrieved from SRA and mapped to *P. trichocarpa* genome v3.0 using a similar approach as described by Sundell et al (Sundell et al., 2017). Briefly, quality of the reads was assessed using FastQC (http://www.bioinformatics.babraham.ac.uk/projects/fastqc/ v0.11.9), then residual adapters and low quality reads were trimmed using Trimmomatic v0.39 (Bolger et al., 2014), and filtered reads were mapped to the reference genome using STAR v2.7.6 (with parameters – outFilterMultimapNmax 100; Dobin et al., 2013). Transcript per million (TPM) values were calculated using Stringtie v1.3.4 (with parameter -M 0.75; Pertea et al., 2015). Significant Pearson correlations with False Discovery Rate (FDR) adjusted *p*<1e-3 were retained for each pair of genes. For each potential candidate gene identified by the GWAS, the top 500 most highly co-expressed genes (highest absolute rand FDR <1e-3) were selected, and GO enrichment was performed considering the GO annotation of the first BLAST hit in *Arabidopsis* of the *P. trichocarpa* gene (*P. trichocarpa* genome annotation file v3.0, available at Phytozome V12), using the R package TopGO. GO terms with FDR adjusted *p ≤* 0.05 were considered significantly enriched and reported in the results.

Protein alignment of AT2G25680 (AtMOT1), Potri.006G245900 (PtrMOT1-2) and Potri.006G246000 (PtrMOT1-1) were generated using the online multiple sequence alignment tool Clustal Omega (https://www.ebi.ac.uk/Tools/msa/clustalo/; Madeira et al., 2022) with default parameters, and visualized using Jalview (Waterhouse et al., 2009).

## 3 Results

### 3.1 Comparisons of LIBS, NAA, and ICP-MS platforms for elemental profiling of poplar samples

In order to identify the most suitable platform (i.e., the ability to detect a wide range of elements, high throughput, and accurate) for the profiling of mineral elements in poplar leaf samples of a large natural population, a subset of 25 samples (out of 584 samples) was analyzed using three different methods and results were compared.

**Figure 1:**
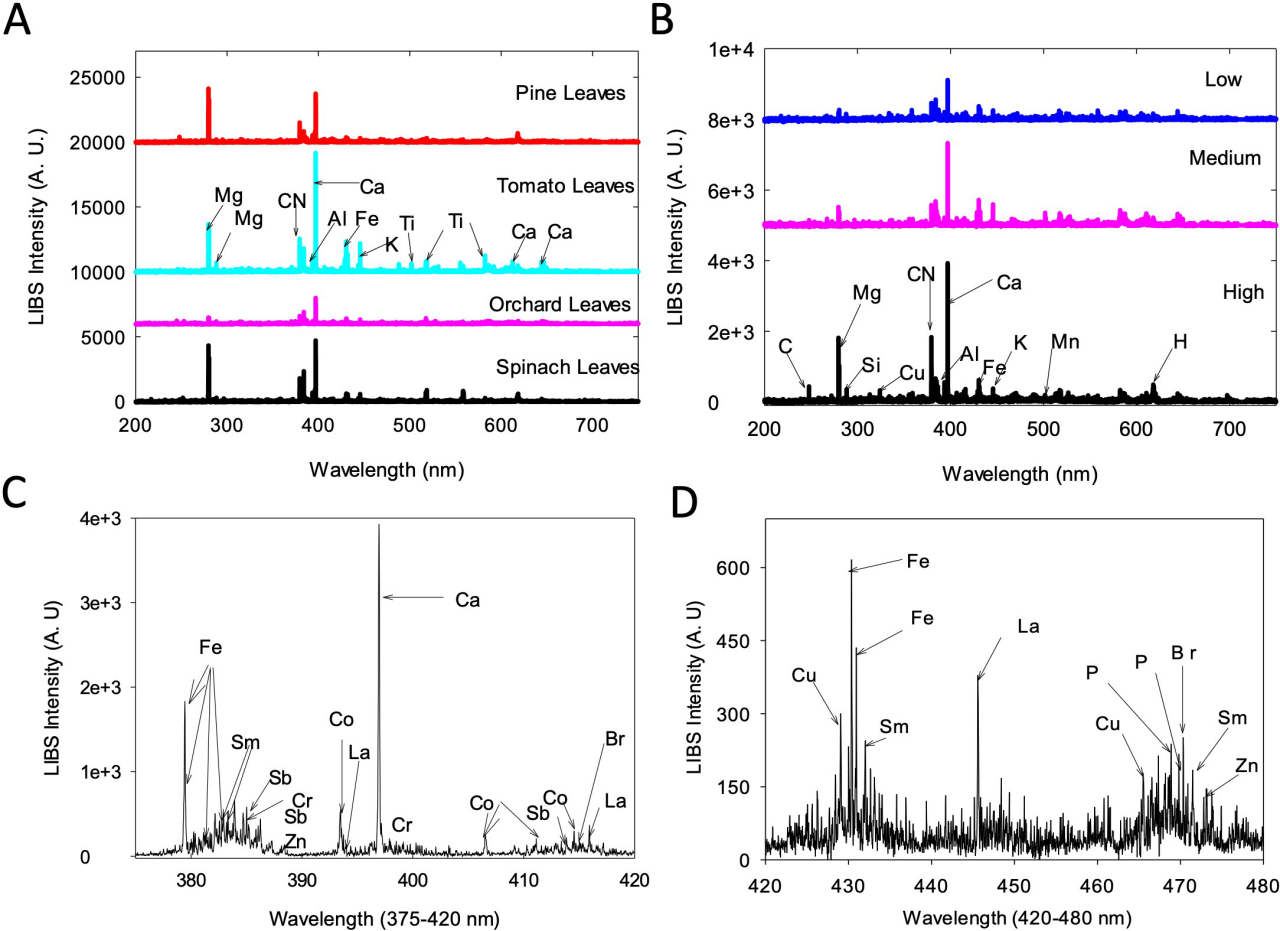
LIBS spectra obtained for NIST standards and poplar leaf samples. **(A)** LIBS spectra for four NIST standards, **(B)** LIBS spectra for 3 poplar samples with low, medium, and high (top to bottom) concentrations of elements, **(C)** LIBS spectrum for a poplar leaf sample (high concentration) that shows 9 minor elements present in the sample in a narrow spectral window of 375-420 nm and **(D)** the detection of copper in the window 420-480 nm. A.U, Arbitrary unit.

Under our conditions, LIBS spectra were able to differentiate elemental peaks among the four NIST standards (**Figure 1A**). Based on these results a variety of major elements were detected in the spectra of the 25-member subset of poplar leaves. Elemental concentrations varied among the tested genotypes, as exemplified in **Figure 1B** with profiles of three genotypes that showed low, medium, and high levels of certain elements.

Results of quantification of Ca, Mg, Mn, Al, Cu, and K from poplar leaves through NAA were highly correlated to what were obtained through LIBS (Martin et al., 2017). However, the LIBS method was able to detect a suite of minor elements that were not available with the NAA method, including Sm, Sb, Cr, Co, La, and Br (**Figure 1C**), with Cu additionally detected in the window 420-480 nm (**Figure 1D**).

**Figure 2:**
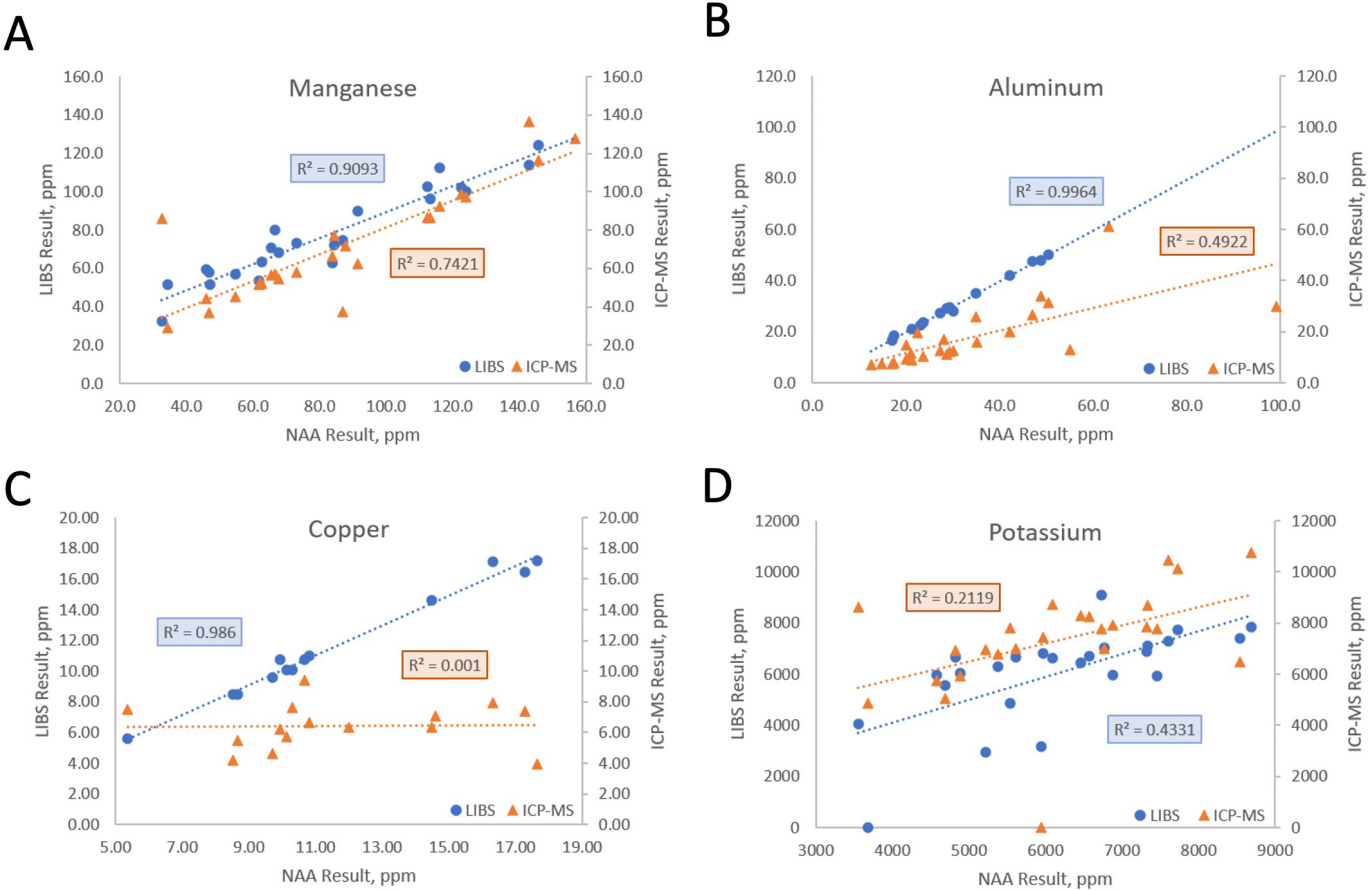
ICP-MS and LIBS comparison to NAA for Mn (A), Al (B), Cu (C), and K (D). Each point represents values obtained using either NAA and LIBS, or NAA and ICP-MS, for the same sample. The coefficients of determination (r^2^) reflect the level of agreement between values obtained from different analytical platforms for these four elements quantified in 25 poplar leaf samples.

We compared the NAA results to both LIBS and ICP-MS for trace and matrix elements that were common to the three techniques (**Table 1**; **Figure 2**). The profiles of several elements showed high correlation between all three methods (e.g., Mg, Ca, Mn; r^2^>0.7, **Figure 2A**), indicating that these elements are relatively homogeneous in the samples and that the three tested platforms perform equally well (**Table 1**). Al and Zn quantification using ICP-MS and NAA showed lower correlation (r^2^ = 0.49; **Figure 2B**, **Table 1**), while quantifications for Cu, Na, and Ru were poorly correlated for these two methods (r^2^ ranging from 0.001 to 0.130; **Figure 2C**, **Table 1**). The results obtained for K were also poorly correlated across methods (**Figure 2D**), suggesting that sample preparation and the method itself affect the quantification of mineral elements.

**Table 1:** Correlation between quantifications obtained using NAA, ICP-MS, and LIBS and reactions used for NAA quantification of the elements.

Element	NAA/ICP-MS Correlation, r^2^ (*p*-value)	NAA/LIBS Correlation, r^2^ (*p*-value)	NAA Reaction^*^
Mg	0.762 (2.61E-08)	0.776 (1.36E-08)	^26^Mg (n,γ) ^27^Mg
Ca	0.882 (1.11E-11)	0.894 (1.06E-12)	^48^Ca (n,γ) ^49^Ca
K	0.395 (1.01E-03)	0.351 (2.30E-03)	^41^K (n,γ) ^42^K
Na	0.034 (0.510)	0.764 (9.51E-06)	^23^Na (n,γ) ^24^Na
Mn	0.742 (6.42E-08)	0.909 (2.04E-12)	^55^Mn (n,γ) ^56^Mn
Cu	0.001 (0.911)	0.986 (1.51E-11)	^65^Cu (n,γ) ^66^Cu
Al	0.492 (1.32E-04)	0.996 (4.90E-16)	^27^Al (n,γ) ^28^Al
Fe	0.687 (3.41E-06)	0.984 (1.72E-19)	^58^Fe (n,γ) ^59^Fe
Zn	0.491 (2.81E-04)	0.990 (1.09E-22)	^64^Zn (n,γ) ^65^Zn
Co	0.831 (3.75E-09)		^59^Co (n,γ) ^60^Co
As	0.778 (1.62E-03)		^75^As (n,γ) ^76^As
Rb	0.130 (0.099)		^85^Rb (n,γ) ^86^Rb

^*^Superscript numbers represent isotope number of the elements.

### 3.2 Elemental profiling of a population of poplar natural variants and GWAS

**Figure 3:**
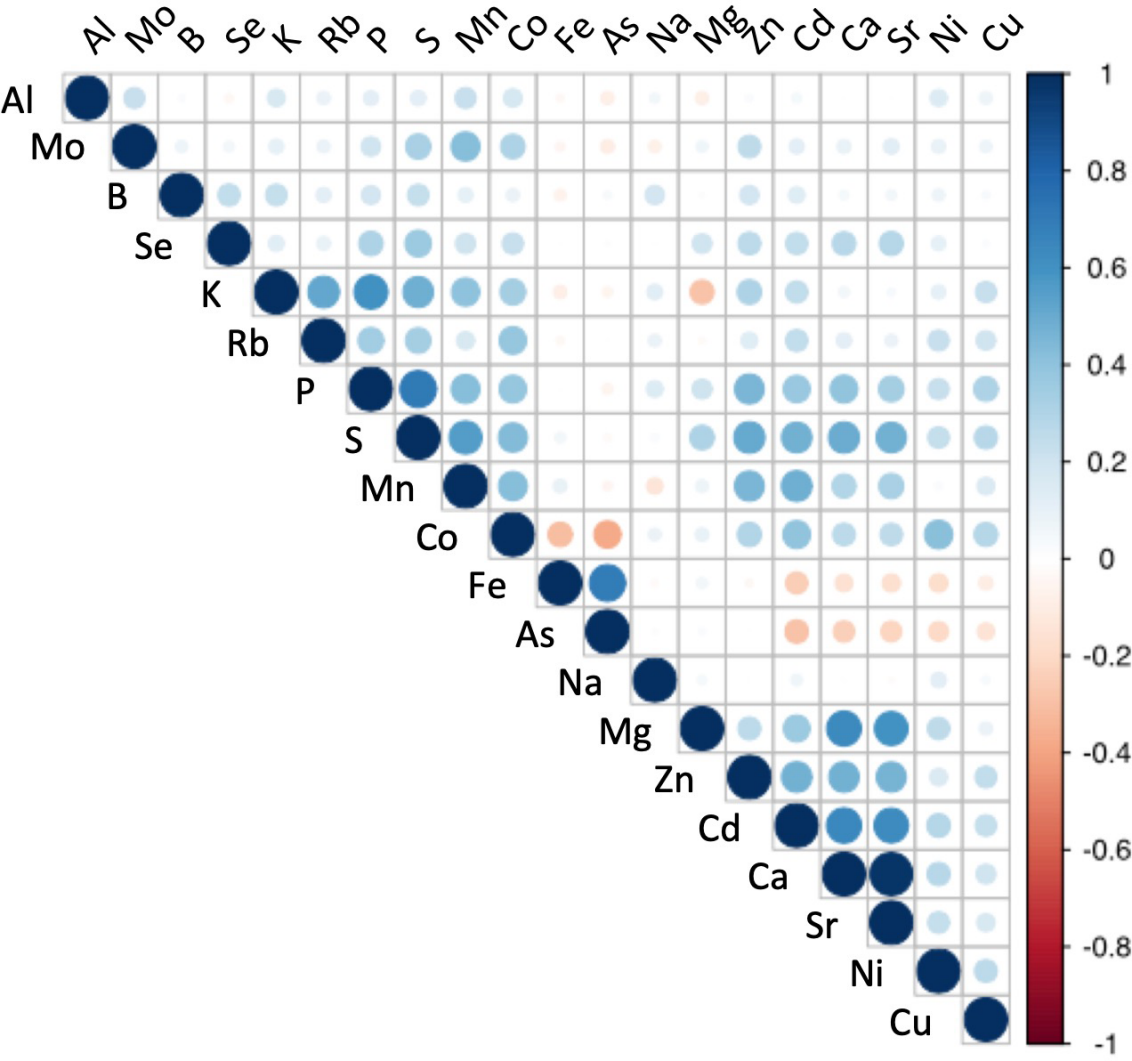
Correlation analysis for leaf elemental composition across 584 *Populus trichocarpa* genotypes. Pearson pairwise correlation coefficients were calculated from the profile of each element across the 584 individual genotypes.

Since the ICP-MS method had a broad range of detectable elements, a relatively high throughput, and showed significant correlation with other methods for most elements, we used the IPC-MS method to characterize the profile of 20 elements (**Figure 3**) across the 584 genotypes of the population. We observed significant variation in the content of all the elements. Interestingly, macronutrients essential for plant growth (P, K, Ca, and Mg) had relatively smaller standard deviations, ranging from 12 to 27% within the population, than those of most micronutrients (Fe, Zn, Cu, Mn, B, Ni, and Mo), which varied between 32 and 50% (**Supplementary Table S2**). Other trace elements, such as Cd, As, and Co, also showed larger variation than macronutrients (relative standard deviation ranging from 35 to 45%), which is consistent with observations from previous studies in other species (Yang M. et al., 2018).

Eight elements exhibited high positive correlation in their abundances, suggesting a possible shared genetic regulation and/or interactions in common biological processes. These elemental pairs had significant Pearson correlation values greater than 0.60 (*p ≤*0.05; **Figure 3**) and included: Ca/Sr (r=0.97, *p* < 2.2e-16), P/S (r=0.72, *p* < 2.2e-16), As/Fe (r=0.69, *p* < 2.2e-16), Ca/Cd (r=0.65, *p* < 2.2e-16), Ca/Mg (r=0.64, *p* < 2.2e-16), Cd/Sr (r=0.63, *p* < 2.2e-16), Mg/Sr (r=0.60, *p* < 2.2e-16) and K/P (r=0.60, *p* < 2.2e-16).

The GWAS was performed to test associations between >8.2 million SNPs and profiles of 19 elements across 584 individual genotypes. Since boron had a distribution that significantly deviated from a normal distribution, it was not considered in the GWAS (**Supplementary Figure S3A, B**). The GWAS performed on the ICP-MS generated ionomic profiles, identified 631 SNPs significantly associated with at least one of the 19 elements at a threshold of -log10 (*p*) >6 (**Supplementary Table S3**; **Supplementary Figures S4** and **S5**). Of the 19 elements, Al, Cu, and Na accounted for 69% of all associations detected, including associations with a total of 434 unique SNP positions (207, 114, and 113 SNPs, respectively), suggesting that Al, Cu, and Na content are highly polygenic traits in poplar (**Supplementary Table S4**). Since the comparison of the analytical methods ICP-MS/NAA revealed the potential for more technical variability in the estimation of these three mineral elements, only the strongest associations were considered for further investigation, by considering the top 100 SNPs with lowest *p*-value. Across all other elements, an average of 13 SNPs were significant; for P, K, Ca, and Mg, 3 to 18 SNPs were found significantly associated with variations in macronutrients content (**Supplementary Table S4**).

### 3.3 Genetic determinants of trace elemental composition

**Figure 4:**
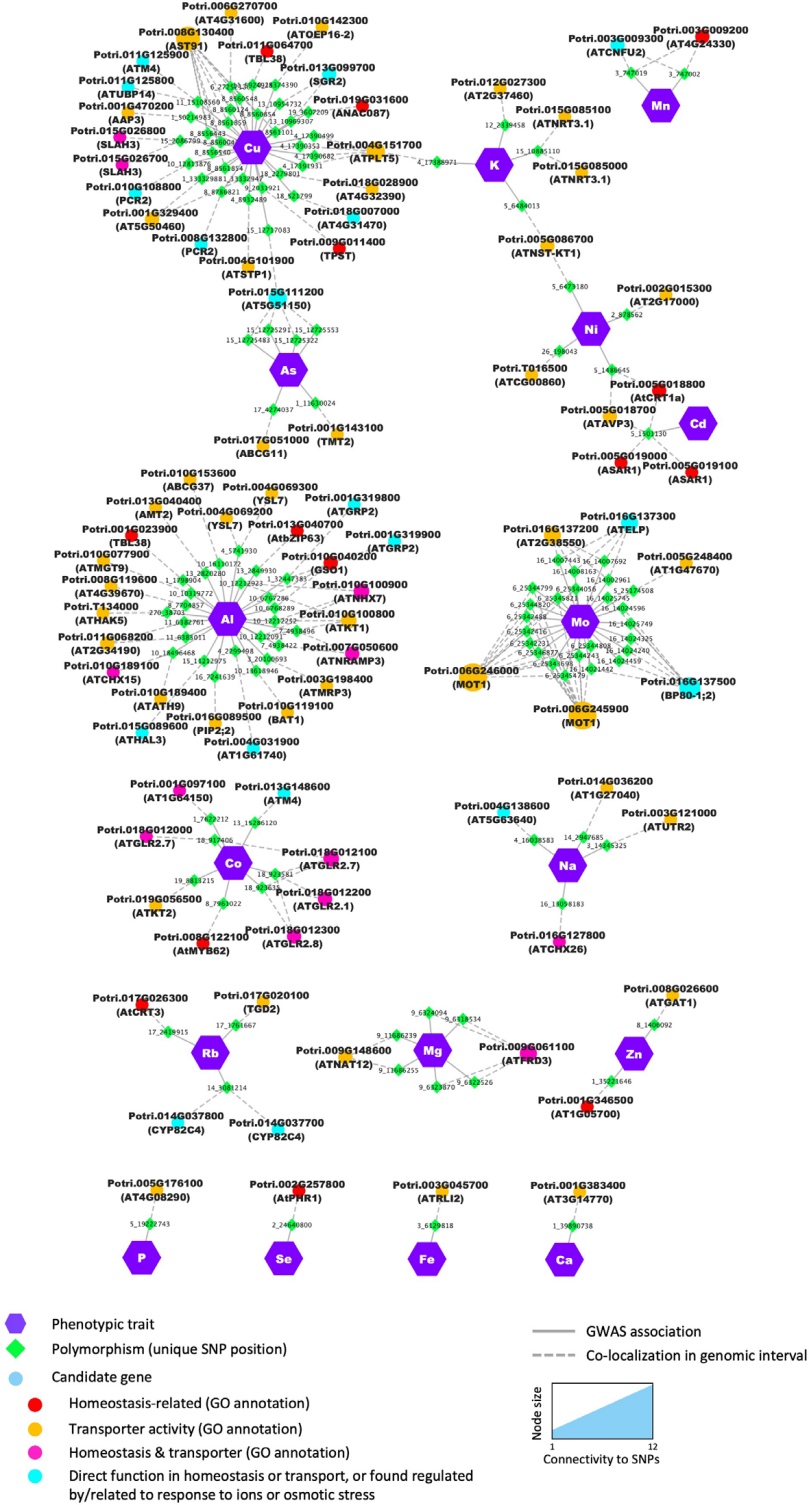
Candidates identified through GWAS were significantly enriched in genes from functional categories related to ion homeostasis and/or with transport molecular function. Nodes (i.e., circles and polygons) represent either phenotypic traits (mineral elements), polymorphisms (unique SNP positions) or potential candidate genes detected in the genomic interval flanking the significant SNP. For each candidate gene, the gene name of the best BLAST hit is provided in brackets in addition to the poplar accession number. When no *Arabidopsis* name was available, the gene accession number in *Arabidopsis* was provided. Edges (i.e., lines) represent the significant association detected by the GWAS (i.e., solid lines) or the colocalization of the SNP and the annotated gene in the genomic interval (i.e., dashed lines). For candidate genes, node color annotates the different functional categories and molecular functions.

The 497 SNPs that passed the statistical threshold were associated with 922 potential candidate genes flanking these positions. Among these, 26 genes were putative homologs of *Arabidopsis* genes annotated within at least one of the biological process GO terms ‘multicellular organismal-level chemical homeostasis’ (GO:0140962), ‘intracellular chemical homeostasis’ (GO:0055082), ‘inorganic ion homeostasis’ (GO:0098771), ‘monoatomic ion homeostasis’ (GO:0050801), ‘sequestering of metal ion’ (GO:0051238), ‘regulation of cellular localization’ (GO:0060341) and ‘regulation of sequestering of calcium ion’ (GO:0051282), which represents a significant enrichment of 1.53 for these categories (*p* =0.0132). Notably, 18 of the 922 genes had a putative *Arabidopsis* homolog annotated within the biological process GO term ‘inorganic ion homeostasis’ (GO:0098771), giving a significant enrichment of 1.61 (*p* =0.0187) (**Figure 4**, **Supplementary Table S5**). When filtering the GWAS results at a more stringent threshold on SNP *p*-value (i.e., -log10(*p*)>8), enrichment in genes annotated in that GO term ‘inorganic ion homeostasis’ increased to a value of 2.29 (*p* =0.0428), strongly suggesting that the GWAS was able to capture relevant candidate genes that control variations in elemental concentrations in poplar leaf tissues.

Notable candidate genes examples include members of the cation–proton antiporters (CPA) family previously shown to regulate cation and pH homeostasis in plants (Chanroj et al., 2012; Khan et al., 2018). For Na and Al several candidates in the CPA family included Potri.016G127800 and Potri.010G189100, putative homologs of *Arabidopsis* AtCHX26 and AtCHX21/23 (Chanroj et al., 2012), respectively, and Potri.010G100900, homolog of the Na+/H+ exchanger AtNHX7/SOS1 (CPA1) shown to be essential for salt tolerance in *Arabidopsis* (Wu et al., 1996) (**Figure 4**, **Supplementary Table S5**). Potri.015G085000 and Potri.015G085100 homologs of the *Arabidopsis* high-affinity nitrate transporter AtNRT3.1 (Kawachi et al., 2006) were found associated with K content, and Potri.010G108800 and Potri.008G132800, potential homologs of AtPCR2 involved in zinc transport and detoxification in *Arabidopsis* (Song et al., 2010), were found associated with the micronutrient Cu content.

Additionally, 55 genes had their *Arabidopsis* homologs annotated with the molecular function GO term ‘transporter activity’ (GO:0005215; **Figure 4**). Although the proportion of genes discovered from that category over the number of genes expected by chance was favorable (enrichment of 1.21), it was not statistically significant (*p* =0.0634), suggesting that although several transporters appear to control elemental composition, as expected, other classes of proteins must be involved in ion homeostasis in poplar leaf tissues. In addition, there were a number of genes predicted to be involved in the direct control of ion fluxes across membranes, including several transcription factors such as Potri.008G122100, homolog of AtMYB62, involved in the response to P starvation in *Arabidopsis* (Devaiah et al., 2009).

**Figure 5:**
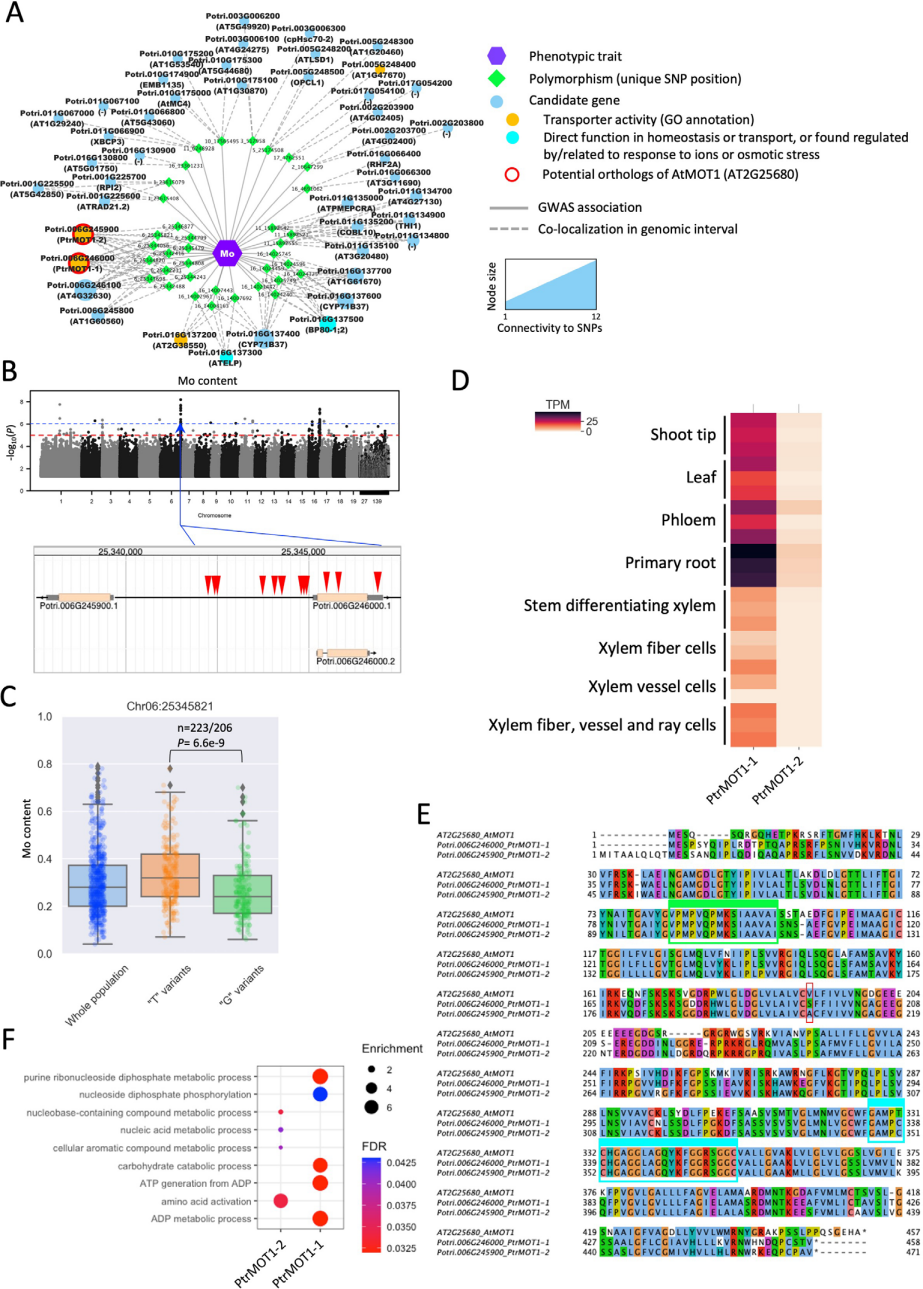
Detection of MOT1 as a determinant of Mo content in the leaves of poplar. **(A)** Results of the GWAS for Mo content – all candidates identified by significant SNPs at a threshold of -log10(*P* value)>6 are represented and the two potential orthologs of AtMOT1 (Potri.006G246000 – PtrMOT1-1 and Potri.006G245900 – PtrMOT1-2) are highlighted in red (nodes represent either Mo content, SNP positions or potential candidate genes identified in the vicinity of the SNPs; edges represent a GWAS association or colocalization of the SNP and the annotated gene in the genomic interval). **(B)** Manhattan plot for the Mo content (blue dotted line indicates the threshold for considering SNPs significant) and the genomic positions of the SNPs found significantly associated with variations in Mo content on chromosome 6. **(C)** Difference in accumulation of Mo between the two groups of genotypes carrying either the T or G allele at position 25345821 on chromosome 6 – significance was assessed using a two-sided Student’s t-test. **(D)** Protein alignment of *Arabidopsis* AtMOT1 (AT2G25680) and the two putative orthologs (PtrMOT1-1 and PtrMOT1-2), showing a conservation of the two domains previously found conserved in MOT1 across Eukaryotes and Prokaryotes (highlighted in green and blue). **(E)** Transcript levels (RNA-seq) of PtrMOT1-1 and PtrMOT1-2 across different tissues of *Populus trichocarpa*. **(F)** GO-BP enrichment results for the top 500 genes co-expressed with PtrMOT1-1 and PtrMOT1-2.

Among the candidate genes with transporter activity, one striking example is the detection of orthologs of the molybdenum transporter AtMOT1 associated with variations in Mo content in poplar leaves. Eleven genomic intervals were found significantly associated with changes in Mo content, containing 44 candidate genes, of which 6 are predicted to have a transporter activity or can be related to ion homeostasis (**Figure 5A**). On chromosome 6, twelve contiguous SNPs associated with variations in Mo were detected in the vicinity of PtrMOT1-1 (Potri.006G246000) and PtrMOT1-2 (Potri.006G245900), which share homology with a known molybdenum transporter AtMOT1 (Tomatsu et al., 2007; Tejada-Jiménez et al., 2007; Baxter et al., 2008b; Tejada-Jiménez et al., 2013). Nine SNPs were detected in the promoter region (295 to 2884 bp upstream the ATG start codon) and 3 SNPs were detected in the coding sequence (2 in the single exon and one in the 3’ UTR) of PtrMOT1-1 (**Figure 5B**). At position Chr06:25345821, the missense mutation was associated with significant differences in accumulation in Mo, with the variants carrying the G allele and having a significantly lower Mo content than the variants carrying the T allele (**Figure 5C**). PtrMOT1-1 harbored the highest transcript level across the 8 poplar tissues, in comparison to its paralog PtrMOT1-2, with a preferential expression in the roots, suggesting that PtrMOT1-1 is the functional copy of MOT1 in *P. trichocarpa* (**Figure 5D**). Interestingly, the mutation at position Chr06:25345821 (highlighted in red in the alignment in **Figure 5E**) caused the substitution of S (reference sequence) by an A in the protein sequence of PtrMOT1-1, which is identical to the sequence of its paralog MOT1-2, which does not appear to be the dominant copy of MOT1 in poplar. When considering the top 500 closest neighbors of PtrMOT1-1 in the co-expression network, this copy of MOT1 was found co-expressed with genes significantly enriched in genes involved in primary metabolism, which is consistent with previous reports on the function of AtMOT1 as cofactor for enzymes involved in several essential biological processes (Peng et al., 2018; **Figure 5F**).

Next, to identify which biological processes underlie variations in elemental concentrations in poplar leaves, we relied on a co-expression analysis and the principle of ‘guilt-by-association’ to infer the biological function of some of the potential candidates. A co-expression approach was taken to identify the genes that are the most highly co-expressed with the candidate genes identified for each element and the main biological functions enriched in that group of genes was determined using a GO enrichment test.

**Figure 6:**
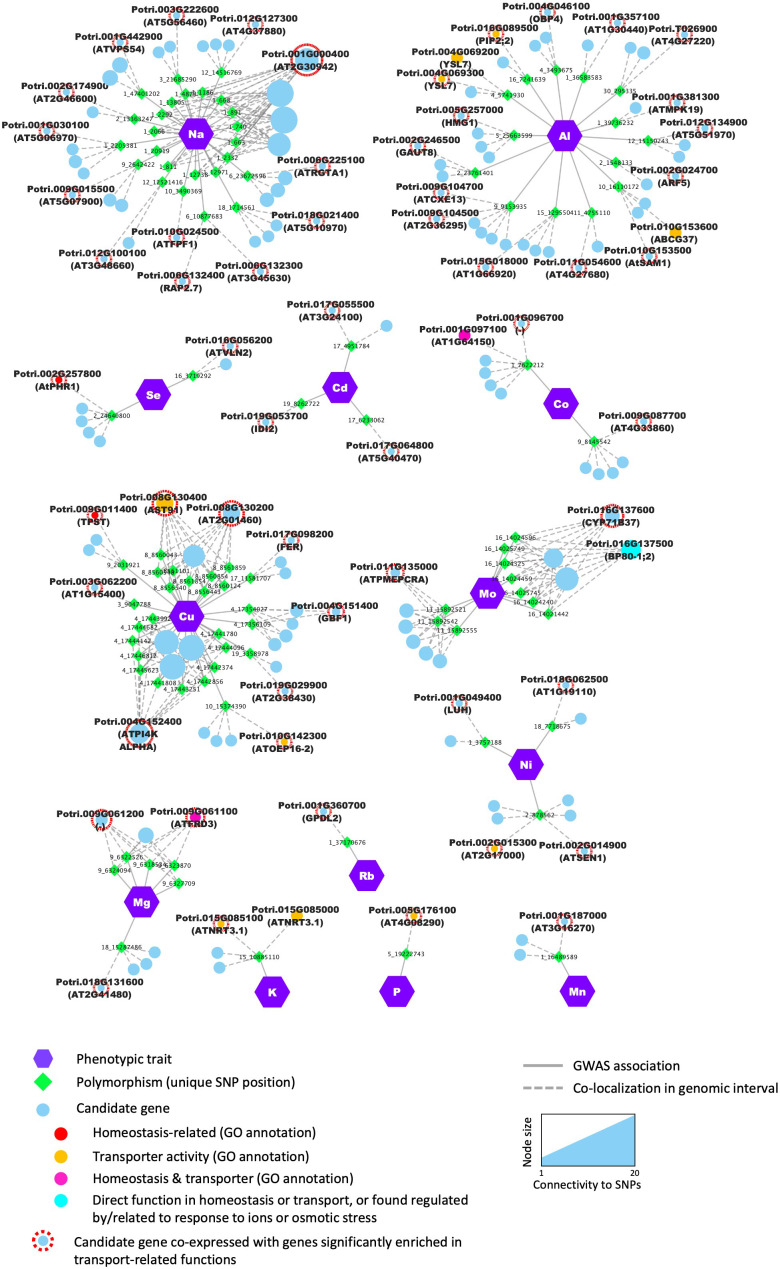
The GWAS identified candidate genes that are co-expressed with genes involved in ion homeostasis and transport. Nodes (i.e., colored circles and polygons) represent either phenotypic traits (i.e., mineral elements), polymorphisms (i.e., unique SNP positions) or potential candidate genes detected in the genomic interval flanking the significant SNP. Enrichment in genes related to transport among the co-expressed genes was tested using a GO enrichment analysis. Candidate genes which were co-expressed with genes enriched in transport-related GO terms are highlighted with a red outline. The exact number of co-expressed genes considered for GO enrichment for each candidate is provided in **Supplementary Table S4**. Edges (i.e., lines) represent significant associations detected by the GWAS or the colocalization of the SNP and the annotated gene in the genomic interval.

Candidates were found to be co-expressed with genes enriched in numerous biological functions (**Supplementary Table S5**), with an over-representation in photosynthesis-related, transport-related, and cytoskeleton-related GO terms. Notably, 57 candidate genes showed a significant enrichment in multiple transport-related GO terms, including 10 that have transport activity or are involved in chemical homeostasis according to molecular function and biological process GO categories annotations (**Figure 6**, **Supplementary Table S5**). For a total of 96 SNP positions, at least one of the flanking genes in the genomic interval was supported by significant co-expression with genes related to transport (**Figure 6**, **Supplementary Table S5**). Examples include multiple genes related to P starvation response in *Arabidopsis* such as Potri.002G257800 (AtPHR1), Potri.008G130400 (AtAST91) and Potri.002G014900 (AtSEN1), that we found associated with micronutrient and trace-element content in poplar leaves. Potri.015G085100, the homolog of the nitrate transporter AtNRT3.1 was also found to be co-expressed with numerous genes enriched in functions such as ‘ion transport’, ‘cation transport’ and ‘transmembrane transport’ (**Figure 6**).

The two candidate genes Potri.004G069300 and Potri.010G182200, were picked from two distinct loci associated to variations in Al content in our conditions (**Figure 6**, **Supplementary Table S5**). Potri.004G069300 was found to be co-expressed with genes that showed a significant enrichment in the GO term ‘transmembrane transport’, which is consistent with its predicted function given that it is a putative homolog of AtYSL7 that belongs to a family of metal-nicotianamine transporters that contributes to uptake and partitioning of metals in plant tissues (Curie et al., 2009; Waters et al., 2006). Interestingly, Potri.010G182200, a putative homolog of ATWI-12/SAG20, a senescence associated gene in *Arabidopsis* (Weaver et al., 1998), was found to be co-expressed with genes related to nicotianamine biosynthesis (**Supplementary Table S5**). Nicotianamine is an important molecule involved in the uptake, delivery, and circulation of metals in plants (Takahashi et al., 2003; Curie et al., 2009).

The SNP at position Chr09:6324094, was associated with variations in Mg and is located in the 3’ region of Potri.009G061100, a MATE efflux family protein found to be co-expressed with genes related to ‘ion transport’, ‘cation transport’, and biotic stress response (**Supplementary Table S5**). The *Arabidopsis* ortholog of this gene, Ferric Reductase Defective 3/Manganese Accumulator 1 (FRD3/MAN1, AT3G08040), has been reported in numerous studies related to elemental accumulation in plant including iron (Delhaize, 1996; Rogers and Guerinot, 2002) and zinc (Pineau et al., 2012), with a role in biotic stress response (Scheepers et al., 2020). Together these results demonstrate that our GWAS was able to identify several genomic regions that contain genes known to be involved in the control of the ionome in plants, as well as a number of genes not yet reported to be involved in these processes.

**Figure 7:**
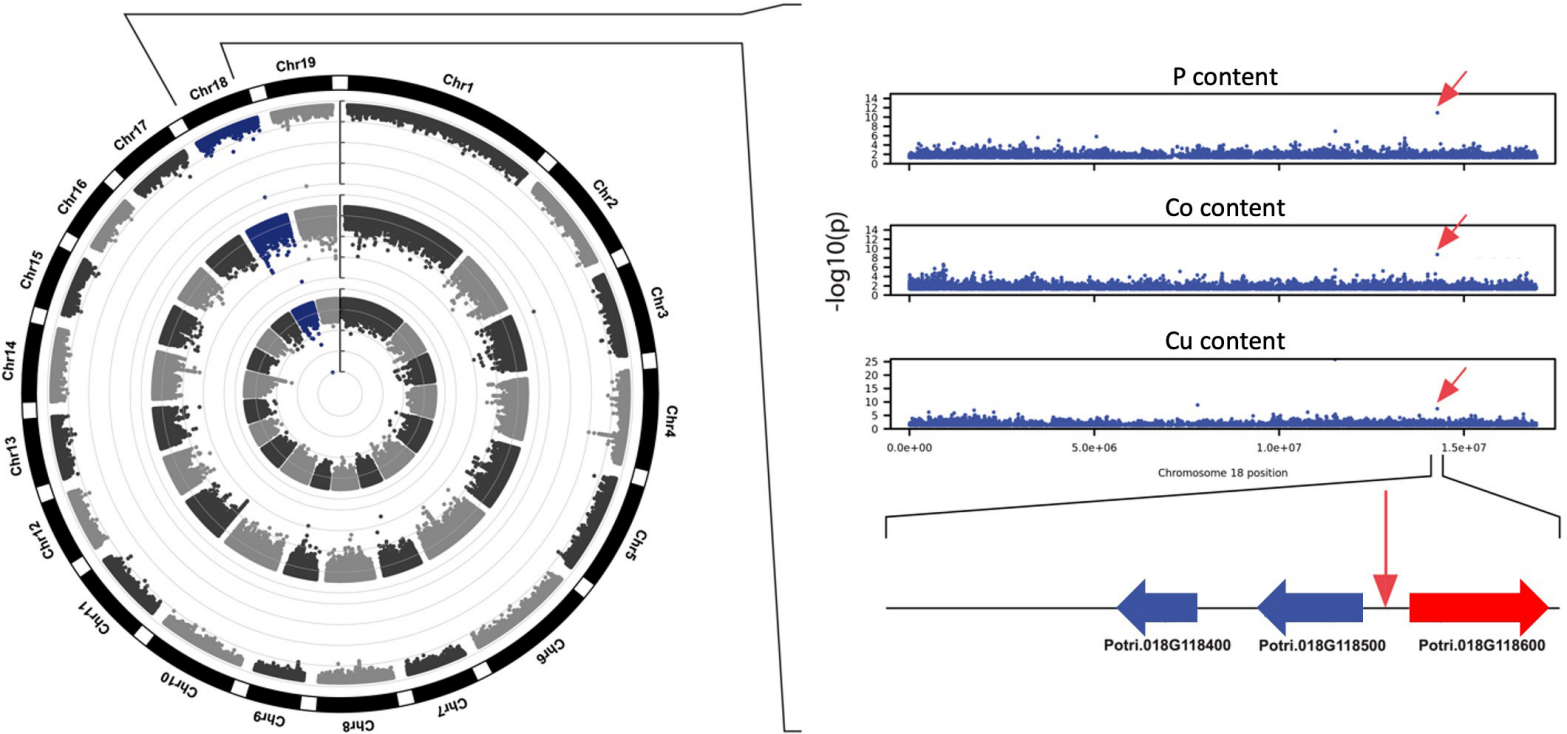
Circular Manhattan plot showing a genomic region on chromosome 18 exhibiting significant associations with abundance of elements P, Co, and Cu. Genome-wide associations detected for elements P, Co, and Cu are represented in the exterior, middle and interior rings of the circular Manhattan plot, respectively. Candidate gene Potri.018G118600 (red) was previously shown to be upregulated under phosphorus limitation.

Across all GWAS associations, six SNP positions were significantly associated with variations in more than one element. The most significant association for the macronutrient P was for the SNP located on Chromosome 18, position 14277823, which exhibited the lowest *p*-value for that phenotype (*p*=1.20E-11) and was also associated with two other elements: Co (*p*=1.96E-09) and Cu (*p*=3.58E-08; **Figure 7**). This genomic interval harbors three candidate genes: Potri.018G118400, encoding a leucine-rich repeat family protein, Potri.018G118500, encoding a homolog of the *Arabidopsis* protein S-acyl transferase AtTIP1, and Potri.018G118600, predicted to encode a 2-oxoglutarate and Fe (II) dependent oxygenase superfamily protein. Interestingly, the *Arabidopsis* homolog of Potri.018G118600, AT1G49390, was reported to be systemically regulated under phosphorus starvation (Thibaud et al., 2010), suggesting that Potri.018G118600 could underlie the association observed in this genomic interval. Our GWAS results also suggest that Potri.018G118600 could play a role in the homeostasis of and/or in the response to Co and Cu in poplar.

**Figure 8:**
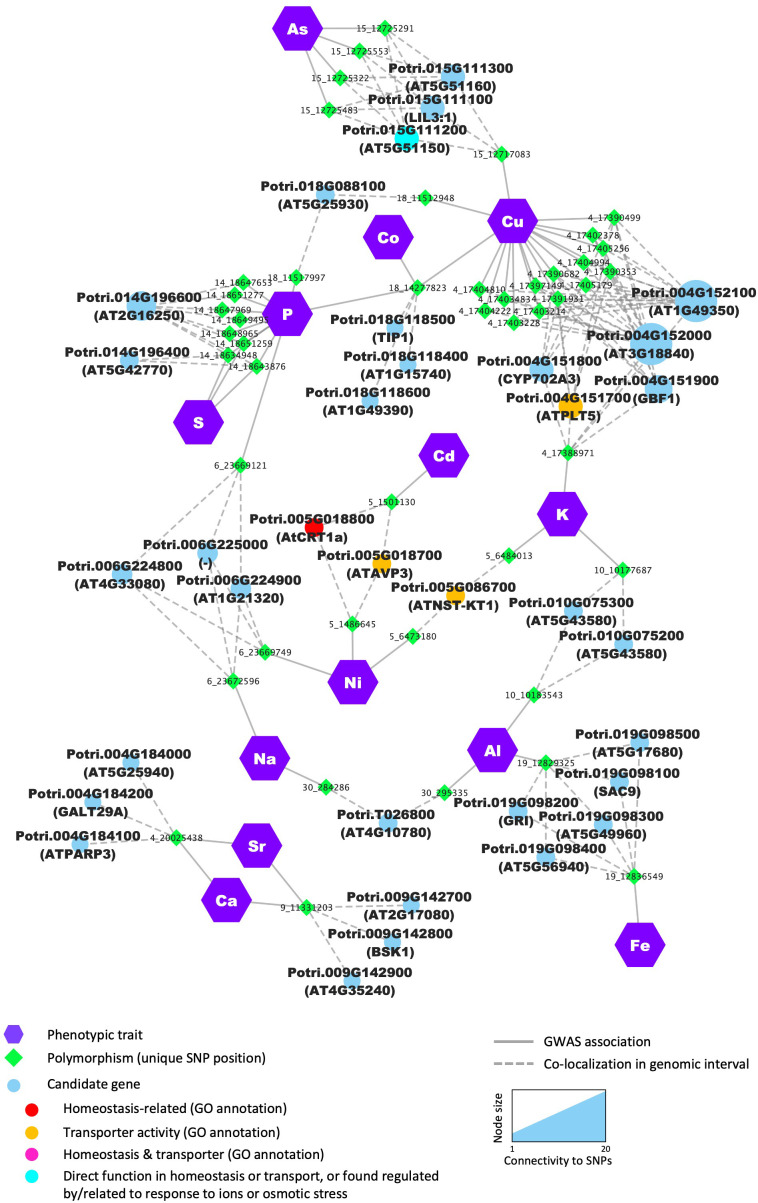
Several candidate genes are detected as determinants for multiple elements. Nodes (i.e., colored circles and polygons) represent either phenotypic traits (elements), polymorphisms (unique SNP position) or candidate genes detected in the genomic interval flanking the significant SNP. For candidate genes, node color indicates the different functional categories. Edges (i.e., lines) represent the significant association detected by the GWAS or the colocalization of the SNP and the annotated gene in the genomic interval.

Multiple SNP positions located in two genomic intervals were associated with Ca and Sr content (**Figure 8**). On chromosome 4, the most promising candidate is Potri.004G184000, shares homology with *Arabidopsis* AT5G25940, which was identified by Schmidt et al. as a potential vacuolar membrane protein (Schmidt et al., 2007). P content was associated with 8 SNPs, all located in the same genomic interval on chromosome 14, of which 3 were also associated to S content. All SNPs pointed to one potential candidate, Potri.014G196600, predicted to encode a leucine-rich repeat protein kinase family protein with no clear function attributed yet (**Figure 8**; **Supplementary Table S5**). These results suggest the existence of shared genetic regulation between elements that are essential nutrients for plant growth, and our approach uncovers several potential key players in this cross-homeostasis in poplar leaf tissues.

In total, 34 genes were found associated with multiple mineral elements. These genes were identified based on SNPs directly associated with multiple mineral elements or located in the overlap between genomic intervals of different SNPs associated with multiple mineral elements, including 5 genes previously associated with ion homeostasis and/or with transporter activity, such as Potri.005G018800 and Potri.005G018700 both identified by two SNP positions found associated with the two heavy metals, Ni and Cd (**Figure 8**; **Supplementary Table S5**). Potri.005G018800 is a homolog of AtCRT1a, a member of the calreticulin protein family, localized in the ER and involved in multiple cellular processes, including calcium homeostasis (Christensen et al., 2010). The second candidate, Potri.005G018700, is predicted to encode a H+-pyrophosphatase, homologous to *Arabidopsis* vacuolar pyrophosphatase AVP1. The function of AVP1 has been extensively characterized in multiple plant systems and has been shown to contribute to cation uptake and sequestration, with AVP1 overexpressing plants showing different levels of accumulation of K and Na in leaves and increased resistance to high NaCl or Mg and low P (Gaxiola et al., 2001; Yang et al., 2007, 2015). Interestingly, to date, none of these gene families have been directly related to the response to heavy metals.

In addition, Potri.004G151700, homologous to the broad substrate-specificity H^+^-symporter AtPMT5 in *Arabidopsis* (Klepek et al., 2010, 2005), was associated with both K and Cu content (**Figure 8**; **Supplementary Table S4**). Potri.004G151700 was found highly co-expressed with genes related to photosynthesis in our co-expression analysis, consistent with a critical role of K and Cu as macro- and micro-nutrients that impact photosynthetic processes. Several genes were identified as potential major regulators of ion partitioning for multiple elements. That was the case for Potri.018G088100, which was related to two elements critical for photosynthesis (P and Cu; **Figure 8**) and was co-expressed with genes related to photosynthesis and primary metabolism (**Supplementary Table S5**). Other candidates were co-expressed with genes enriched in various biological processes, which highlights the tight connection between the elemental composition in the leaves and overall poplar physiology (**Figure 8**; **Supplementary Table S5**).

In order to determine the extent genetic architecture controls of the ionome is conserved between a tree species and other plants, we tested the overlap between candidates found in our study and previous ionomics studies coupled with GWAS in rice and *Arabidopsis* (Yang M. et al., 2018). Despite differences in the experimental procedures and the limitations of orthology inference, 34 of the genes we detected as candidates in our study had potential orthologs associated to variations in elements in rice by Yang M. et al., (2018) (**Supplementary Table S5**). Notably, Potri.016G089500, predicted to encode a plasma membrane intrinsic protein subfamily PIP2, was associated with variation in Al and co-expressed with transport and ion homeostasis-related genes in poplar, and its potential ortholog (LOC_Os07g26660) was associated with variations in N content in rice (**Supplementary Table S5**; Yang M. et al., 2018). Furthermore, 7 overlapping candidates were also detected with the ionomics study that authors performed in *Arabidopsis*. Among these, the poplar gene Potri.T134000, a homolog of AtHK5, a high affinity K+ transporter in *Arabidopsis* (Rubio et al., 2008), was associated with Al content in our study and with Mo content in *Arabidopsis* (Yang M. et al., 2018). In addition to these results, the systematic detection of polymorphisms associated to Mo content located in the promoter and/or coding region of MOT1 across all three species (**Supplementary Data S5**), suggests a degree of conservation in the genetic architecture that controls variation in the ionome across plant species. Interestingly, most of the candidates overlapping across studies have not been related to ion homeostasis yet (**Supplementary Data S5**).

## 4 Discussion

During their life cycle, plants uptake mineral ions that are considered macronutrients, micronutrients, or trace elements. The uptake, transport, and storage of these ions within and across tissues are crucial processes that impact plant physiology. The paucity or excess amounts of some of these nutrients and trace elements can have detrimental effects on plant growth, leading to reduced yield, reduced fitness, and/or increased sensitivity to abiotic and biotic stressors (Pandey, 2015). Improving agronomic traits requires an understanding of how the ionomic profile varies and which genes mediate such variation. Several gene families are known to have conserved roles across plant species in these processes, including transporters, channels, and chelators (Aibara and Miwa, 2014); yet the genetic determinants of ionome variations remain largely unknown.

In numerous plant species, the use of natural genetic diversity has been used in forward genetics screening for identifying genes and regulatory mechanisms that underlie biological functions and determine a phenotype. By directly pointing at the genomic loci and the causal mutations, GWAS have been instrumental at identifying genes that are responsible for variations in many traits of interests in poplar (Muchero et al., 2015; Tuskan et al., 2018b; Xie et al., 2018; Zhang et al., 2018; Chhetri et al., 2020; Bryant et al., 2023; Nagle et al., 2023; Saint-Vincent et al., 2023). One major challenge for setting up this type of approach is the need to phenotype large populations (typically in the order of hundreds of individuals), with a method that can quantify a broad spectrum of elements with high sensitivity to capture slight variations across individuals and high accuracy.

Here we investigated three different platforms (LIBS, NAA, and ICP-MS) for the profiling of mineral elements in poplar leaf samples of a large natural population. All three methods agreed for several elements, however results for the quantification of K showed low correlation. Other elements such as Cu, Al, Na and Zn also showed relatively poor correlations between ICP-MS and NAA. This might be largely due to major differences in sample preparation, but it is also intrinsically linked to the analytical methods themselves. For NAA and LIBS analyses, pellets were pressed after humidification, while this pre-processing was not required for ICP-MS. The LIBS and NAA samples were pretreated by humidification which led to slightly different sample density which is manifested as weighing differences. Regarding the difference in the analytical methods, LIBS detects mineral elements on particles or small individual fragments of tissue (from the regions of the pellets targeted by the laser pulses), which makes it sensitive to heterogeneity in the sample composition. In the contrary, IPC-MS and NAA work on larger amounts of material, which tends to make these methods less sensitive to tissue heterogeneity, although ICP-MS analyses the sample after digestion, which is fundamentally different from NAA that analyses the sample in its native form (ground tissues in that case). The low correlations among the three methods for K, compared to the other elements, suggests that K content may not have been uniform across the tested samples. The higher abundance in K in certain cell types, such as in the guard cells of the stomata for example, might contribute to variations in content measured through LIBS and NAA. Alternatively, ICP-MS values for K should be more accurate since a larger sample mass was dissolved for analysis than that taken for LIBS. Overall, NAA, LIBS, and ICP-MS are independent methods of analysis that each exploits different properties of the elements. NAA works on the nucleus and not the valence electrons, LIBS works on the energy level structure of the electron shells, and ICP-MS analyzes elements by ionization and mass separation. NAA is not sensitive to the valence state or chemical compound of the trace elements being measured which makes it less matrix sensitive and independent of chemical measurement methods such as ICP-MS. However, unlike ICP-MS, the detection sensitivity of NAA varies for each element according to the probability of neutron interaction and gamma-ray emission, gamma-ray energy, and decay half-life. These differences between the methods could explain the poor agreement between ICP-MS and NAA for Na.

Given that ICP-MS is the most suited platform to profile a broad range of mineral elements in a relatively high-throughput manner and considering that it has been largely used for ionome profiling in multiple plant systems (Baxter et al., 2012, 2008; Yang M. et al., 2018; Wu et al., 2021), we selected that method to profile the leaf ionome of 584 genotypes of *P. trichocarpa*. The analysis of the ionome of the population revealed that the content in micronutrients (Fe, Zn, Cu, Mn, B, Ni, Mo) and other trace elements (Cd, As, Co) was more variable than that of the macronutrients we quantified (P, K, Ca, Mg). A similar trend was observed in other species (e.g., rice and *Arabidopsis*; Yang M. et al., 2018), supporting the hypothesis that across annual and perennial plants, the concentration of the essential nutrients is under tight genetic control to ensure optimal growth, while micronutrients and trace elements are more prone to fluctuation.

To identify the genetic determinants of mineral ion composition in poplar leaves, we then performed GWAS, testing associations between a set of high-density SNPs (~19 SNP per kb on average) and the elemental profile of 19 elements quantified using ICP-MS. This high-resolution mapping pointed to 922 unique potential candidate genes that could mediate, or be related to, variations in ion composition in the leaf tissues, of which 69 had putative orthologous genes already annotated to ion homeostasis and/or transport in other species. Even though the ionome of poplar leaves is not expected to be controlled exclusively by genes annotated with these GO terms, the significant enrichment in genes related to these biological processes or molecular function was expected and demonstrates that the GWAS captured relevant associations.

Our study provides strong evidence that a number of these candidates are the causal genes for the variation in elements. One ortholog of the high affinity molybdenum transporter MOT1 (Peng et al., 2018) emerges as the strongest candidate in our approach. In previous GWAS performed in *Arabidopsis* (Baxter et al., 2010; Chao et al., 2014, 2012; Forsberg et al., 2015), rice (Yang M. et al., 2018), maize (Wu et al., 2021) and cassava (Jin et al., 2022), MOT1 has been identified as a gene directly related to the Mo content. In our GWAS, one of the copies of MOT1 (PtrMOT1-1) showed a high degree of polymorphism, with several of these polymorphisms located in the promoter and coding region related to variations in Mo content. The fact that MOT1 is systematically identified as a strong candidate for Mo content in many ionomic GWAS suggests that Mo accumulation in plant tissues is highly dependent on MOT1 activity and reflects the level of conservation of that mechanism across species (Tomatsu et al., 2007; Peng et al., 2018), including in a tree species like black cottonwood poplar. The large number of SNP in the 5’ promoter region also suggests that the regulation of MOT1 is high controlled at the gene transcription level and that multiple transcription factors may exist that control transcription.

Another example of a direct association is the detection of PtrCHX16 (Potri.016G127800; Chanroj et al., 2012), a homolog of AtCHX26, as a candidate for Na content. Cation/H+ exchangers family (CHX) proteins belong to the MONOVALENT CATION PROTON ANTIPORTER-2 (CPA2) family of transporters involved in cation transport in plants (Chanroj et al., 2012; Khan et al., 2018). Although the exact function of most members of that family remains unclear, CHX were previously related to K+, Na+ and other monovalent cation transport and homeostasis and were shown to play a role in various cellular and physiological processes, including pH regulation, membrane trafficking, osmoregulation for stomatal opening and pollen tube growth (Cellier et al., 2004; Hall et al., 2006; Padmanaban et al., 2007).

Interestingly, another member of the CPA2 transporter family, PtrCHX25 (Potri.010G189100; Chanroj et al., 2012), a homolog of AtCHX21/23, together with Potri.010G100900 homolog of the Na^+^/H^+^ exchanger AtNHX7/SOS1 that belongs to the CPA1 transporter family, were associated with variations in Al content in poplar leaves. AtCHX21/23 was related to K transport, osmoregulation, and pollen tube growth, while extensive evidence demonstrated the role of AtNHX7/SOS1 in salt stress tolerance and K homeostasis (reviewed in Chanroj et al., 2012). This is the first report of this gene family being directly related to Al content. Similarly, the gene PtrNRT3.1C (Potri.015G085100; Plett et al., 2010) belongs to the NRT3 family and contributes to high affinity NO_3_^-^ transport in *Arabidopsis* (Plett et al., 2010), while it was found associated to K content in our study. Whitt et al. (2020) compiled a list of genes experimentally shown to change uptake, accumulation, and distribution of mineral elements across different species. Our analytical and computational approach related 9 genes of that list to variations in several elements in poplar (**Supplementary Table S5**). Except for the two copies of MOT1 that we related to Mo content, the 7 remaining genes were found associated to different elements than those reported in previous functional studies. Previous large-scale studies performed on the ionome of *Arabidopsis*, rice and maize (Baxter et al., 2010; Wu et al., 2021; Yang M. et al., 2018), uncovered many similar patterns of associations between an element and a likely candidate gene that was shown to be involved in the transport or homeostasis of another mineral element. Outside of the divergence between monocots and dicots (or annual *Arabidopsis* vs. perennial *Populus* within dicots), where in some cases, these associations can be explained by lineage specific evolutions that led to subfunctionalization of the paralogs, this type of indirect shared association highlights the importance of mineral interactions in plants.

The existence of strong relationships between different nutrients has been shown to underlie agronomic traits and physiological processes in many plant species (Baxter, 2015; Bouain et al., 2019). Typically, changes in content of one element can induce changes in the accumulation of other elements through competition for transport and uptake or by impacting absorption of other elements indirectly by changing developmental processes such as root architecture for example (Kellermeier et al., 2014) or cellular and physiological processes such as pH control and osmotic adjustment (Singh et al., 2013). A notable example is AVP1 in *Arabidopsis*, a pyrophosphatase (H+-PPase) proton pump that controls the electrochemical gradient across the vacuolar membrane. Overexpression of AVP1 improved NaCl tolerance in *Arabidopsis* (Gaxiola et al., 2001) and other plants including wheat (Brini et al., 2005), tobacco (Duan et al., 2007) and poplar (Yang et al., 2015). In *Arabidopsis*, overexpression of AVP1 resulted in enhanced cation uptake with an accumulation of Na and K in the leaf tissues, suggesting that AVP1 promotes sequestration of cations into the vacuole (Gaxiola et al., 2001). Interestingly it also influenced P nutrition, with AVP1 overexpressing *Arabidopsis*, tomato, and rice plants outperforming wild-type plants when grown with limited P. Overexpression of one of the related genes (PtVP1.1) in poplar also led to higher growth and resistance to NaCl treatment, together with a decreased amount of Na and higher amounts of K in poplar leaves (Yang et al., 2015). *Arabidopsis* plants overexpressing AVP1 also showed higher magnesium tolerance Yang Y. et al., 2018) as one of the phenotypes.

Many other examples of cross-homeostasis between nutrients exist in the literature, especially between the macronutrients N/P/K and metals or trace elements, such as Fe, Zn, S, Cu, Co, Mg and Rb (Baxter et al., 2008a; Bouain et al., 2019; Kocourková et al., 2020; Oliferuk et al., 2020). The significant correlation between profiles of multiple elements detected in our study was observed in other species (Neugebauer et al., 2018), which further supports the existence of interactions between mechanisms related to elements uptake and movement. In *Arabidopsis*, the multidrug and toxin efflux (MATE) transporter FRD3 is involved in distributing mineral elements across the plant tissues and has been shown to play an essential role in the cross-homeostasis of Fe and Zn. Mutants that have impaired growth typically accumulate high levels of different metals, such as Fe, Mn, and Zn in root and shoot tissues (Pineau et al., 2012; Roschzttardtz et al., 2011; Scheepers et al., 2020). It is therefore not completely surprising to find one poplar homolog of AtFRD3 associated with Mg content in our study. Other examples involving the pair of monovalent cations K^+^ and Na^+^ are common in the literature, with the well-studied salt-stress response mechanism involving intracellular Ca^2+^ homeostasis and Ca^2+^ signaling in poplar and other species (Polle and Chen, 2015). All these observations reinforce the hypothesis that in attempts to identify regulators of the ionome, combinations of elements are likely to be the actual phenotype to consider for GWAS, rather than individual mineral elements, as proposed by Baxter (2015).

While previous studies highlighted the importance of considering interactions at the physiological, chemical, and genetic levels when studying mineral element homeostasis in plants, these interactions have remained largely unexplored due to their complexity. Our approach provides additional evidence of common genetic, and possibly physiological, mechanisms associated with mineral concentrations in poplar. Even though the potential causal gene for variations in one element was not systematically directly functionally associated with the homeostasis of that element, often, the likely causal gene was found co-expressed with genes involved in specific biological processes, such as photosynthesis, primary metabolism, and other essential biological functions, indicating that some of these elements interact at the physiological level. The identification of genomic loci associated to pairs of elements (i.e., K and Cu, P and Na, or the heavy metals Ni and Cd) illustrates direct genetic interactions between mineral element concentration, and as such, points to multiple new candidate genes potentially involved in the homeostasis of multiple mineral elements in plants.

In addition to numerous candidate genes with known functions in ion homeostasis, the majority of the genes identified through GWAS could not be related to this type of function solely based on annotation and orthology. In fact, 57 of these candidate genes, related to 13 elements in our GWAS, were found co-expressed with genes involved in transport and ion homeostasis, suggesting that some of the candidates identified belong to functional modules involved in elemental homeostasis.

Improving the uptake of certain mineral elements such as macronutrients and certain micronutrients has been shown to have a beneficial effect on plant growth, by positively impacting processes such as photosynthesis and amino acids biosynthesis for example, while also allowing to reduce the input in fertilizers (Ferrante et al., 2017). In addition to improving growth and fitness, accumulation of certain elements in plant tissues leads to higher resistance to biotic and abiotic stresses (Ali et al., 2021). This is the case for Sulfur (S), a constituent of numerous defense compounds such as glutathione, thionins, defensins and phytoalexins. In Arabidopsis, a higher sulfate supply led to a stimulation of sulfur metabolism associated with a reduced susceptibility to the pathogen *Alternaria brassicicola* for example (Kruse et al., 2012). In fact, in lettuce, a previous study found an association between resistance to *Xanthomonas campestris* and the nutrient balances involving N, P, S and other micronutrients (Nicolas et al., 2019). A higher S supply could also reduce the adverse effects of salinity in Barley (Astolfi and Zuchi, 2013) and contributed to improve drought tolerance in Maize (Usmani et al., 2020). Copper (Cu) is another element notorious for its antimicrobial activities. In rice, the pathogen *Xanthomonas oryzae* manipulates the host’s Cu redistribution to create a favorable environment to multiply in the vascular system (Yuan et al., 2010). On the other hand, higher copper levels can lead to higher laccases activity and subsequent increase in radical production, which can lead to breakages in the cell wall polymers and changes in cell wall physico-chemical properties (Printz et al., 2016). Many other examples exist in the literature, with the availability of certain elements having a broad impact on both the development and stress resistance of plants, such as K. Potassium is involved in numerous processes as a cofactor for enzymes, and as an osmolyte. K was shown to play a critical role in drought resistance in multiple species (Santos et al., 2021; Johnson et al., 2022), while also playing a key role in growth, and wood development in trees for example (Langer et al., 2002; Sardans and Peñuelas, 2021). This study identified new targets for manipulating homeostasis of some of these mineral elements with the potential to improve nutrient uptake, nutrient use efficiency, growth properties, defense responses and resistance to abiotic stresses in poplar and other species.

## Data Availability

The original contributions presented in the study are publicly available. This data can be found here: Oak Ridge National Laboratory, https://labkey.ornl.gov/labkey/CBI/Tuskan/PUBLIC_DATA/Elemental%20profiling%20and%20genomewide%20association%20studies%20reveal%20genomic%20variants%20modulating%20ionomic%20composition%20in%20Populus%20trichocarpa%20leaves/project-begin.view?wizard=true.

## Supplementary material

Supplementary Figure 1 Geographical distribution of the black cottonwood natural variants (*P. trichocarpa*) in the native stands. **(A)** Geographical provenance of the 1,089 genotypes encompassing the central portion of the natural range of the species, stretching from 38.8° to 54.3° N latitude from California, USA, to British Columbia, Canada (in blue on the map), and **(B)** of the subset of 584 genotypes considered in this analysis (in pink on the map).

Supplementary Figure 2 LIBS spectra obtained for the four soil samples collected on the site of the common garden used for the GWAS. **(A)** LIBS spectra in the window 190-350 nm, **(B)** 350-460 nm, and **(C)** 580-800 nm.

Supplementary Figure 3 Distribution of the 20 elements across the leaf samples of 584 *Populus trichocarpa* natural variants. **(A)** density plots and **(B)** probability plots of the profiles.

Supplementary Figure 4 Manhattan plots resulting from the GWAS on the profile of 19 elements. B was not considered for the GWAS.

Supplementary Figure 5 Network representation of the results of the GWAS. Nodes (i.e., circles and polygons) represent either phenotypic traits (mineral elements), polymorphisms (unique SNP positions) or potential candidate genes detected in the genomic interval flanking the significant SNP. For each candidate gene, the gene name of the best BLAST hit is provided in brackets in addition to the poplar accession number. When no *Arabidopsis* name was available, the gene accession number in *Arabidopsis* was provided. Edges (i.e., lines) represent the significant association detected by the GWAS (i.e., solid lines) or the colocalization of the SNP and the annotated gene in the genomic interval (i.e., dashed lines). For candidate genes, node color annotates the different functional categories and molecular functions.
