# Supplementary figures and images for: Elemental profiling and genome-wide association studies reveal genomic variants modulating ionomic composition in Populus trichocarpa leaves
Source: Front Plant Sci. 2024 Nov 28;15:1450646. doi: 10.3389/fpls.2024.1450646 (PMC11634625; doi:10.3389/fpls.2024.1450646)

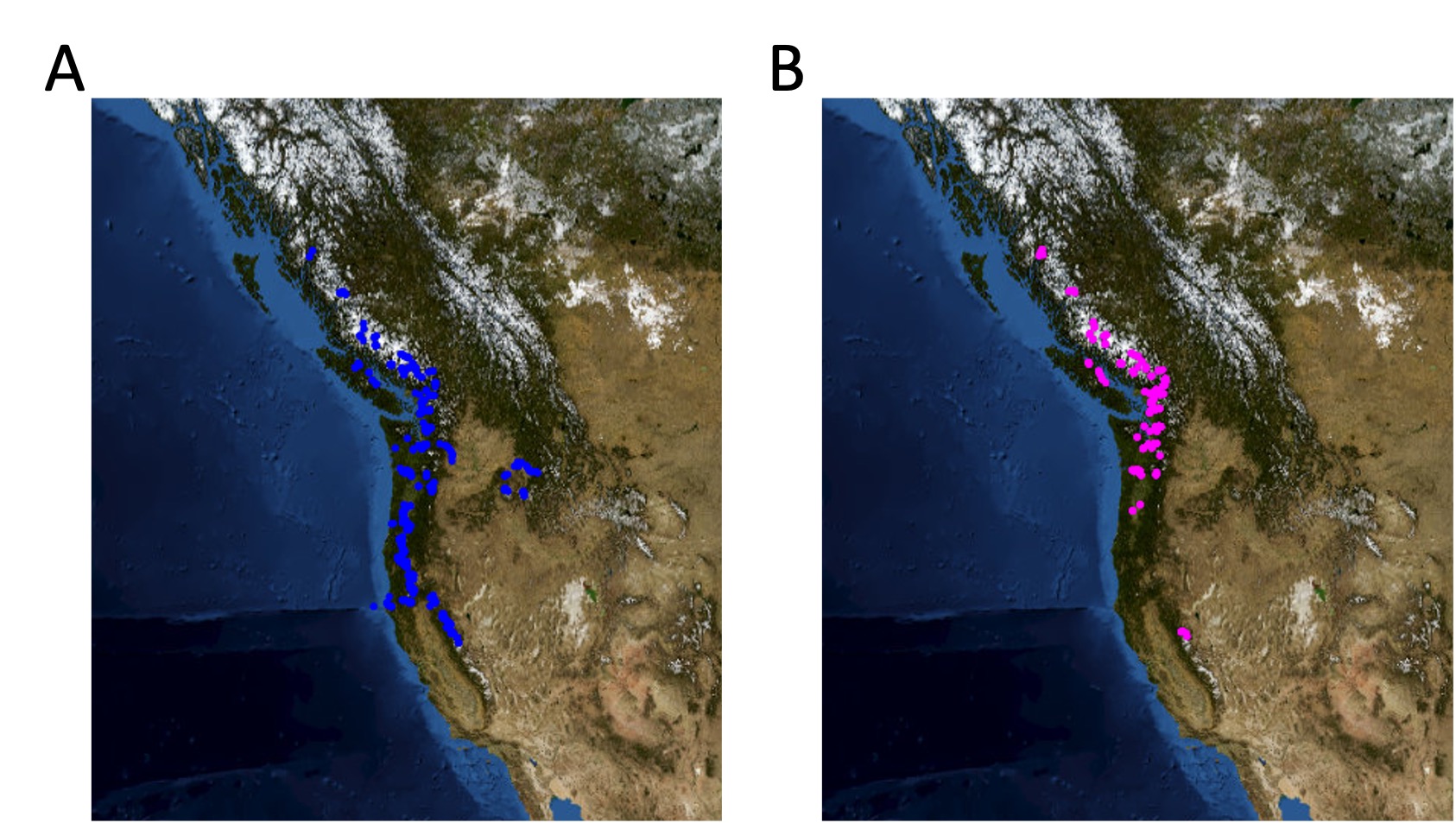

Supplement: Supplementary Figure 1 — Geographical distribution of the black cottonwood natural variants (P. trichocarpa) in the native stands. (A) Geographical provenance of the 1,089 genotypes encompassing the central portion of the natural range of the species, stretching from 38.8° to 54.3° N latitude from California, USA, to British Columbia, Canada (in blue on the map), and (B) of the subset of 584 genotypes considered in this analysis (in pink on the map). [file Image1.jpg]

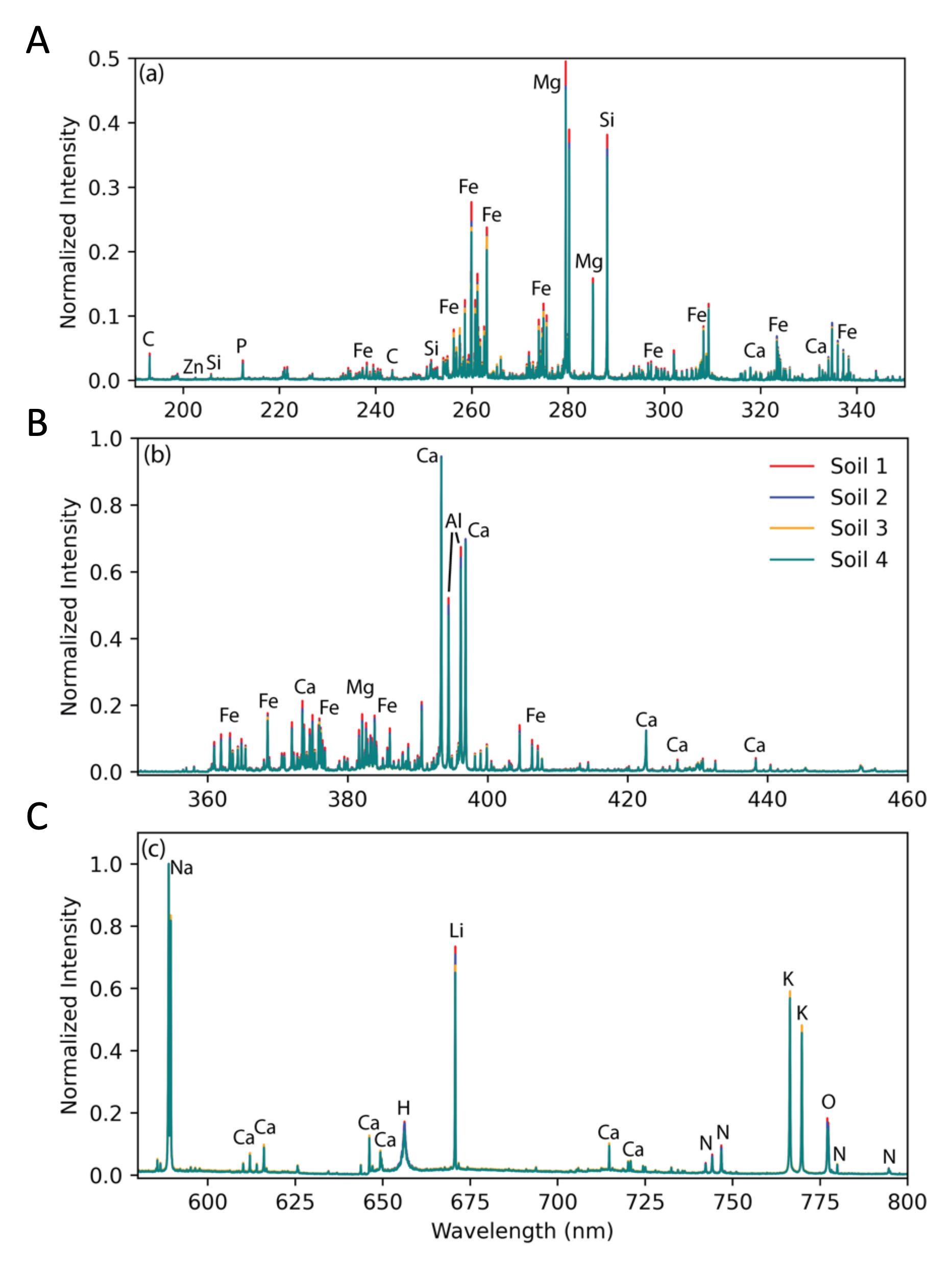

Supplement: Supplementary Figure 2 — LIBS spectra obtained for the four soil samples collected on the site of the common garden used for the GWAS. (A) LIBS spectra in the window 190-350 nm, (B) 350-460 nm, and (C) 580-800 nm. [file Image2.jpg]

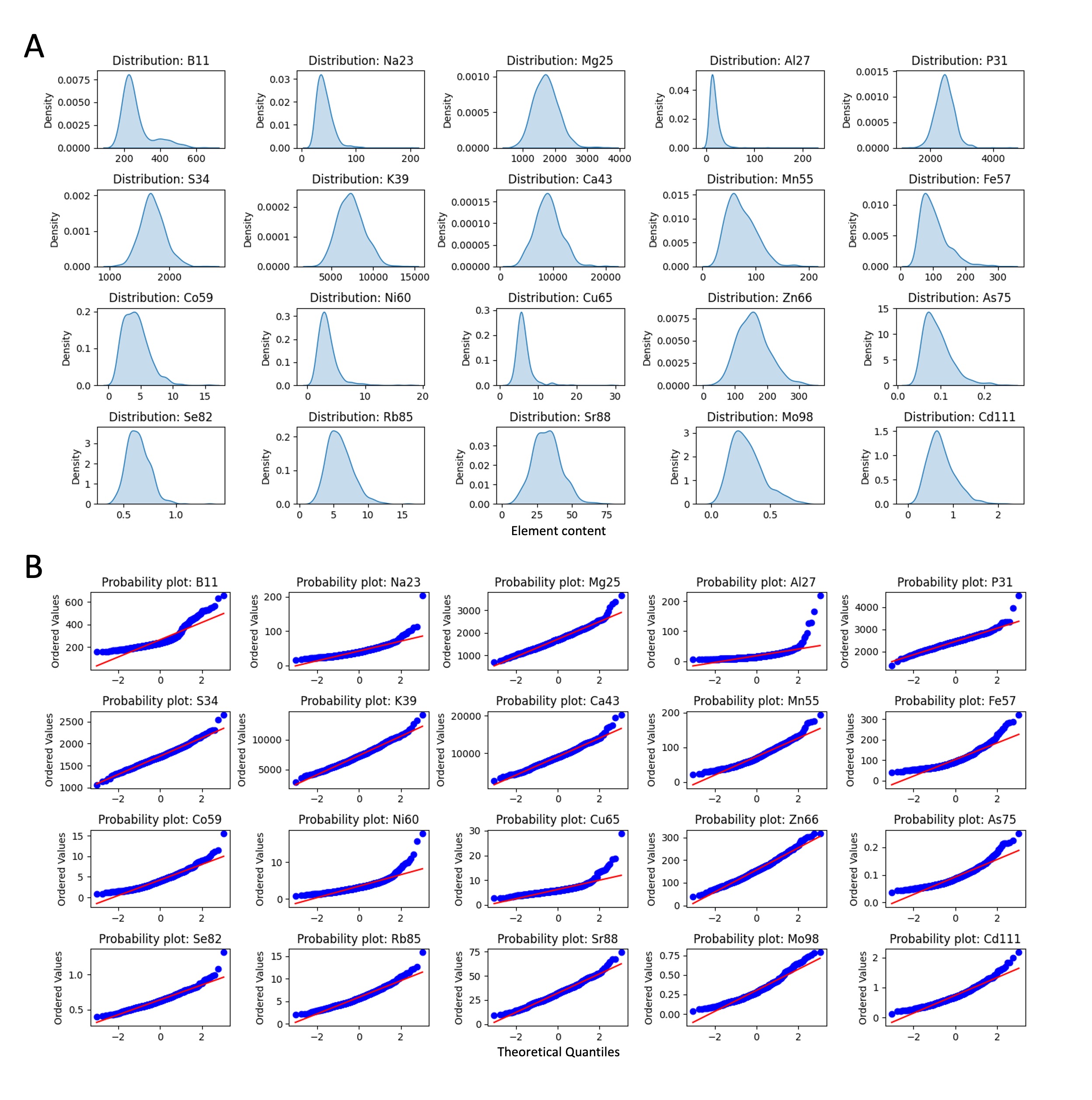

Supplement: Supplementary Figure 3 — Distribution of the 20 elements across the leaf samples of 584 Populus trichocarpa natural variants. (A) density plots and (B) probability plots of the profiles. [file Image3.jpg]

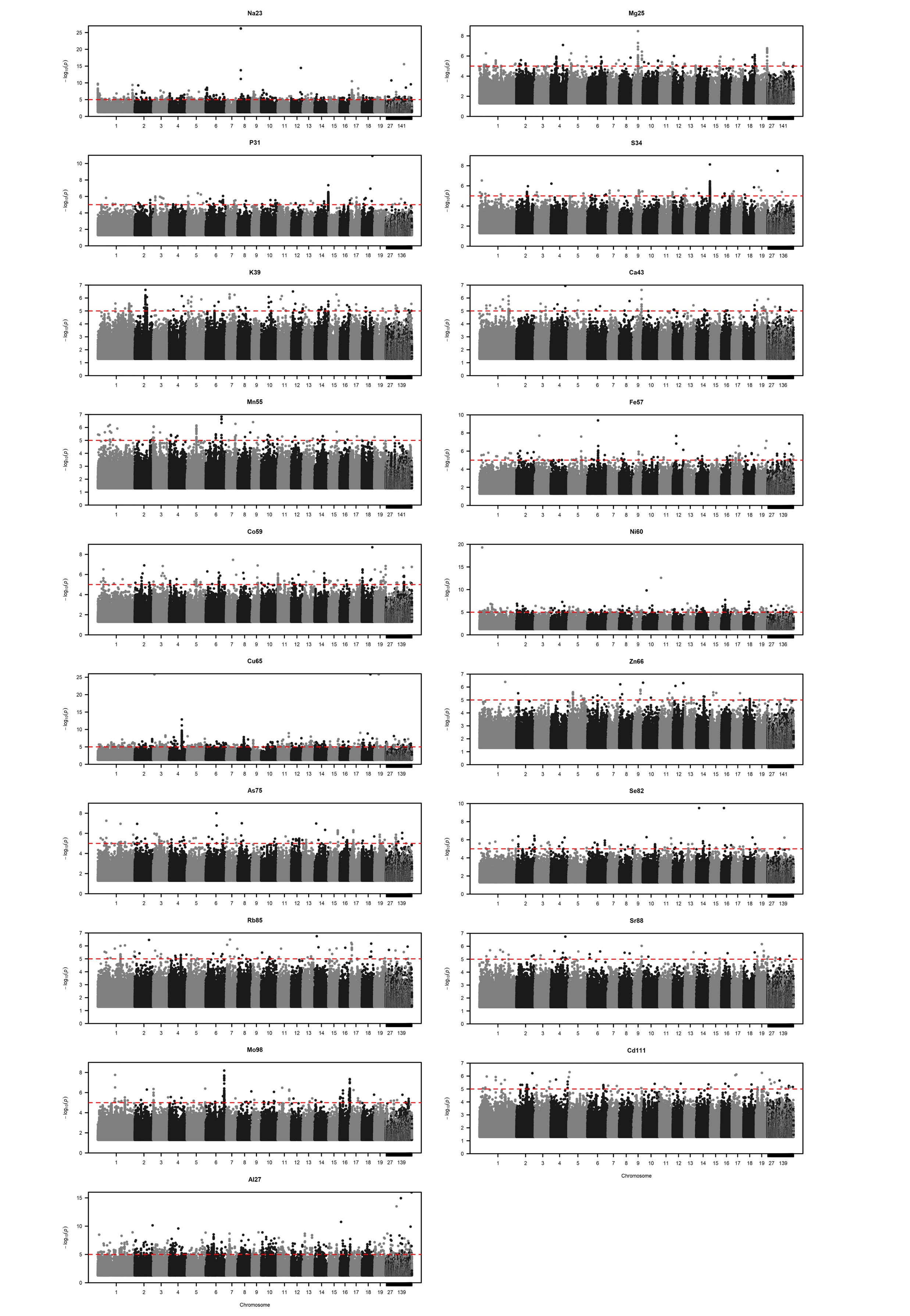

Supplement: Supplementary Figure 4 — Manhattan plots resulting from the GWAS on the profile of 19 elements. B was not considered for the GWAS. [file Image4.jpg]

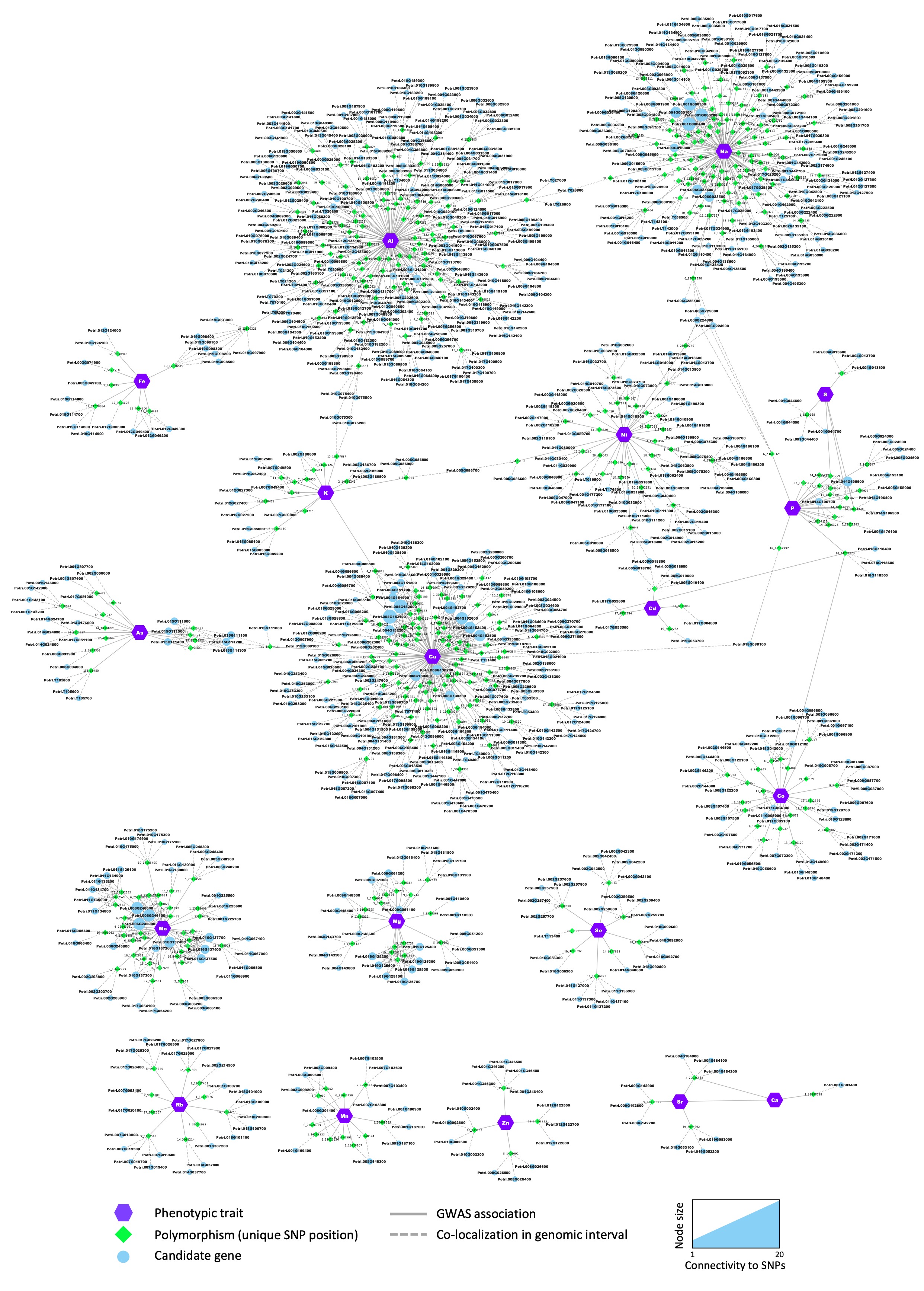

Supplement: Supplementary Figure 5 — Network representation of the results of the GWAS. Nodes (i.e., circles and polygons) represent either phenotypic traits (mineral elements), polymorphisms (unique SNP positions) or potential candidate genes detected in the genomic interval flanking the significant SNP. For each candidate gene, the gene name of the best BLAST hit is provided in brackets in addition to the poplar accession number. When no Arabidopsis name was available, the gene accession number in Arabidopsis was provided. Edges (i.e., lines) represent the significant association detected by the GWAS (i.e., solid lines) or the colocalization of the SNP and the annotated gene in the genomic interval (i.e., dashed lines). For candidate genes, node color annotates the different functional categories and molecular functions. [file Image5.jpg]
